# Common AAV gene therapy vectors show nonselective transduction of *ex vivo* human brain tissue

**DOI:** 10.1016/j.omtm.2025.101494

**Published:** 2025-05-21

**Authors:** JP McGinnis, Joshua Ortiz-Guzman, Maria Camila Guevara, Sai Mallannagari, Benjamin D.W. Belfort, Suyang Bao, Snigdha Srivastava, Maria Morkas, Emily Ji, Angela Addison, Evelyne K. Tantry, Sarah Chen, Ying Wang, Zihong Chen, Kalman A. Katlowitz, Jeffrey J. Lange, Melissa M. Blessing, Carrie A. Mohila, M. Cecilia Ljungberg, Guillermo Aldave, Ali Jalali, Akash Patel, Sameer A. Sheth, Howard L. Weiner, Shankar Gopinath, Ganesh Rao, Akdes Serin Harmanci, Daniel J. Curry, Benjamin R. Arenkiel

**Affiliations:** 1 Department of Neurosurgery, Baylor College of Medicine, Houston, TX 77030, USA; 2 Department of Molecular and Human Genetics, Baylor College of Medicine, Houston, TX 77030, USA; 3 Jan and Dan Duncan Neurological Research Institute, Texas Children’s Hospital, Houston, TX 77030, USA; 4 Medical Scientist Training Program, Baylor College of Medicine, Houston, TX 77030, USA; 5 Development, Disease Models & Therapeutics Graduate Program, Baylor College of Medicine, Houston, TX 77030, USA; 6 Stowers Institute for Medical Research, Kansas City, MO 64110, USA; 7 Department of Pathology, Texas Children’s Hospital, Baylor College of Medicine, Houston, TX 77030, USA; 8 Department of Pediatrics, Texas Children’s Hospital, Baylor College of Medicine, Houston, TX 77030, USA; 9 Department of Neurosurgery, Texas Children’s Hospital, Baylor College of Medicine, Houston, TX 77030, USA

**Keywords:** gene therapy, human brain, AAV, AAV tropism, human brain organotypic slice culture

## Abstract

The ability to deliver a therapeutic sequence to a specific cell type in the human brain would make possible innumerable therapeutic options for some of our most challenging diseases; however, studies on adeno-associated virus (AAV) vector tropism have generally relied on animal models with limited translational utility. For this reason, establishing the tropism of common adeno-associated virus (AAV) vectors in living human brain tissue serves as an important baseline for further optimization, as well as a determination of human brain cell types transduced by clinically approved gene therapy vectors AAV2 and AAV9. We have adapted an *ex vivo* organotypic model to evaluate AAV transduction properties in living slices of human brain tissue. Using fluorescent reporter expression and single-nucleus RNA sequencing, we found that common AAV vectors show broad transduction of normal cell types, with protein expression most apparent in astrocytes; this work introduces a pipeline for identifying and optimizing AAV gene therapy vectors in human brain samples.

## Graphical abstract

**Figure:**
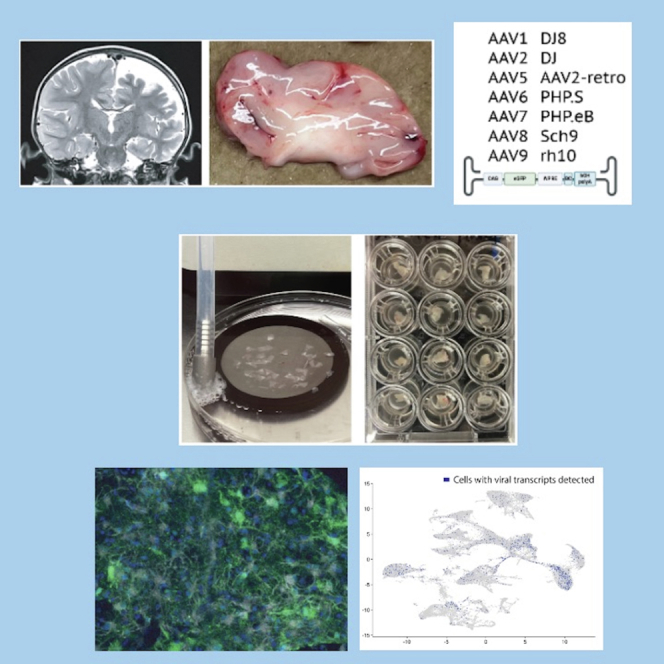

## Teaser

Using *ex vivo* living human brain tissue, McGinnis and colleagues find that common AAV gene therapy vectors nonspecifically label human brain cell types. This suggests that current gene therapies are indiscriminate and highlights the improvements in AAV vectors that could be found screening in human brain tissue.

## Introduction

There is growing recognition that the field of medicine will soon see widespread use of molecular tools, including CRISPR-based gene editing, optogenetics, and other DNA- and RNA-based therapies. These new therapies hold great promise for treating some of our most challenging neurological diseases, from movement disorders and neurodegenerative diseases to epilepsies and tumors.^1^^,^^2^ However, in many cases, the safety and efficacy of these tools will depend on our ability to deliver sequence-based therapeutics precisely to target cell types, while avoiding neighboring but uninvolved cells. Viral-based vectors, including adeno-associated virus (AAV)-derived vectors—the most common vectors in ongoing clinical trials for neurological diseases—rely on their surface proteins’ recognition of cellular membrane components to gain entry to a cell.^3^^,^^4^ For this reason, modifying AAV capsids in order to target specific cell types (that is, modifying an AAV’s ‘tropism') has been an area of broad interest.

Past efforts to screen and develop new AAV capsids for cell-type specificity have relied on animal models or cell lines,^5^^,^^6^^,^^7^^,^^8^^,^^9^ and the limited postmortem human data following intraparenchymal AAV injection trials have not focused on identifying the cell types transduced.^10^^,^^11^^,^^12^ We therefore sought to use living human brain tissue to (1) establish the cellular specificity of Food and Drug Administration (FDA)-approved (AAV2 and AAV9) and other common AAV variants in human brain tissue, identifying any tropism biases that could serve as the basis for iterative approaches to optimization; (2) create a standardized baseline against which to compare future novel capsids; and (3) identify potential species-specific tropism differences that would inform future AAV capsid engineering.

**Figure 1:**
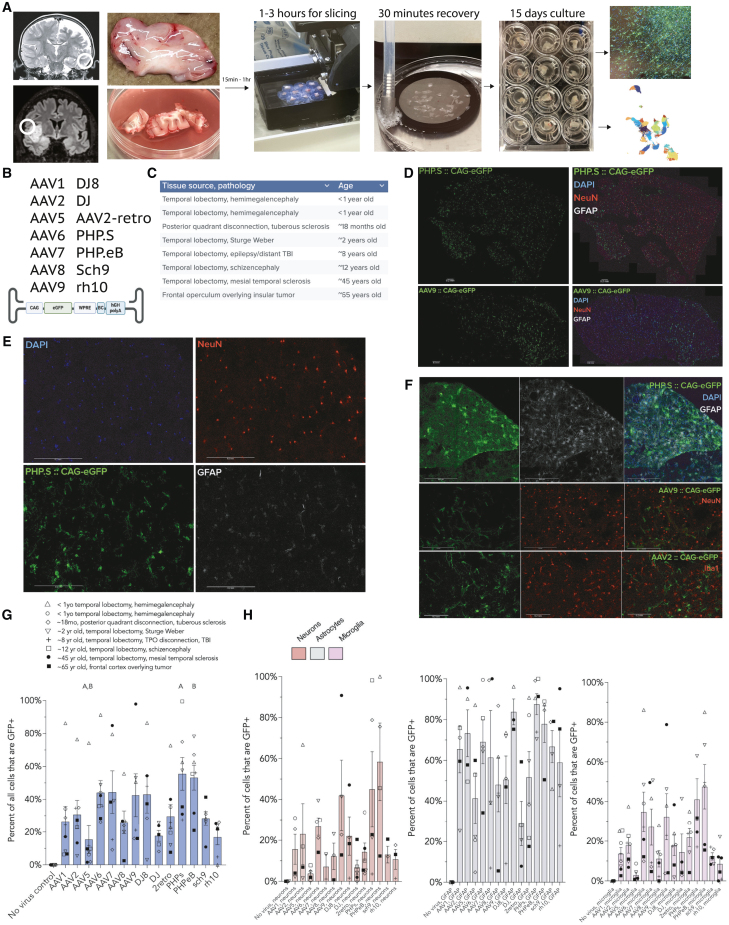
Human brain organotypic slice model facilitates characterization of AAV tropism by immunofluorescence (A) Workflow of the human brain organotypic model: example MRIs with location of tissue resected, resected samples, vibratome slicing, NMDG-aCSF recovery, culture plates, and immunostaining or single-nucleus sequencing. (B) The AAV capsid variants studied and the CAG-eGFP construct used for immunostaining and single-nucleus sequencing. (C) The anatomical locations of patients’ samples, pathologies involved, and approximate ages. (D) Representative images of entire sections of PHP.S and AAV9 from adult frontal cortex overlying tumor. Scale bars: 200 μm. (E) Magnified images of PHP.S with DAPI, NeuN (neurons), GFAP (astrocytes), and transduced cells (GFP). (F) Representative magnified images showing PHP.S and astrocytes, AAV9 and neurons, and AAV2 and microglia in pediatric temporal lobectomy samples. Scale bars: 200 μm. (G) Percent transduction of all cells present in the tissue (measured as percentage of DAPI-based ROIs that show GFP signal higher than the brightest control ROIs). (“A” indicates significant differences from the other bar labeled “A”, as does “B”. Kruskal-Wallis test with Dunn correction for multiple comparisons, adjusted *p* < 0.05). (H) Percentage of NeuN-positive (neurons), GFAP-positive (astrocytes), and Iba1-positive (microglia) cells that co-express GFP, indicating successful transduction of that cell type by the respective capsid variants. Note that the neuronal transduction may be influenced by the loss of neurons over time in culture.

Toward this, we have adapted a human brain organotypic slice model (Figure 1A), using resected neurosurgical specimens not needed for pathologic diagnosis, maintaining the *ex vivo* tissue in a living state for 2 weeks *in vitro.*^13^^,^^14^^,^^15^^,^^16^^,^^17^ We determined the tropism profiles of 14 common AAV vectors via a combination of immunofluorescence and single-nucleus RNA sequencing. We used eight natural AAV variants (AAV1, 2, 5, 6, 7, 8, 9, and rh10) and six engineered AAV variants (DJ8, DJ, AAV2-retro, PHP.S, PHP.eB, and Sch9) (Table S1), all carrying eGFP constructs driven by the ubiquitous CAG promoter. We chose the CAG promoter for its popularity in preclinical and clinical gene therapy studies and its strong and ubiquitous expression pattern. We used brain tissue from eight patients that included temporal lobe cortex and other lobectomy specimens resected due to intractable epilepsies, as well as normal cortex resected en route to deeper tumors (Figure 1C). AAVs were transduced at a uniform titer (2.1E9 vg per tissue slice) established to approximate the viral dose seen by brain cells infused during convection-enhanced delivery trials (Table S2; this is approximately 2E8 vg/mg tissue, though, due to passive diffusion, the effective concentration is heterogeneous through the tissue slice). Reasoning that capsid entry (i.e., AAV DNA presence) is therapeutically useful only to the extent that it leads to productive transcription and translation, we focused on identifying cell types that showed reporter RNA and protein expression 14 days after vector transduction (where vector transduction occurred on post-operative day 1).

## Results

For immunofluorescence analysis, the 300 μm cultured tissue slices were further cryostat-sectioned to 20 μm to facilitate high-resolution imaging. We systematically imaged the two 20 μm sections with the highest GFP expression levels (typically the most superficial sections). Since many slices showed GFP expression eccentrically, we restricted analysis to a 2 mm circle centered around the highest GFP expression for each image to standardize analysis (Figures S1A and S1B) (see materials and methods for detail). These measures were taken with the rationale that we sought to identify each AAV’s transduction efficacy at their presumed point of highest concentration and mitigate the variability that was introduced by differing slice sizes. While this does have the effect of increasing the apparent percentage of cells transduced, it retains the relative relationships between the AAV variants at their point of greatest transduction.

Of the 14 variants tested by immunofluorescence, PHP.eB and PHP.S (55% and 53%, *n* = 7 tissue samples per variant) showed the greatest overall transduction rates (measured as percentage of DAPI-based regions of interest (ROIs) that were also GFP^+^; Kruskal-Wallis test with Dunn post-hoc test) (Figures 1D and 1G). AAV5, DJ, and rh10 showed the lowest transduction rates, all averaging less than 20% of the total cells in the quantified area (*n* = 5–8). AAV2 and AAV9—the most common AAV variants used in neurological trials—both showed moderate transduction, around 30% and 40% of cells within the analyzed area, respectively (*n* = 7 and 6, respectively; for a composite figure of representative images of all capsids, see Figure S2).

For general neuronal transduction, transduction was measured as percentage of NeuN^+^ cells that also expressed GFP; numbers of tissue samples were lower due to some cases where the highest GFP^+^-containing area did not contain sufficient numbers of neurons for evaluation. It should be cautioned that the significant loss of neurons across time may influence our results. With those caveats, PHP.eB and PHP.S showed the greatest transduction rate (∼55% and 45%, *n* = 4 and 5, respectively), with AAV9 and AAV2 transducing ∼40% and ∼30% of NeuN^+^ neurons, respectively (*n* = 4 for each). AAV5 and DJ showed the lowest levels of neuronal transduction (∼5%, *n* = 4 and 5) (Figure 1H). These patterns are notably different from several studies of mouse cellular tropism, which have shown the greatest astrocyte transduction with AAV8 and AAV5 and more prominent neuronal transduction with AAV9.^18^^,^^19^

Astrocytes (GFAP^+^ cells) were notably the most highly transduced cell type for nearly all capsid variants tested. AAV1, AAV2, AAV6, AAV7, DJ8, PHP.S, PHP.eB, and Sch9 all transduced ∼60% or more of astrocytes within the areas of analysis, whereas rh10 and AAV2-retro both transduced just more than 50% of astrocytes analyzed (Figure 1H, *n* = 3–8). The lowest rate of astrocyte transduction in the analyzed areas was observed with DJ and AAV5, ∼30%–40% (*n* = 5–6).

Microglia (Iba1^+^ cells) showed variable transduction, from as low as ∼3%–15% with AAV5, AAV8, and DJ to as high as 45% with PHP.S and PHP.eB (Figure 1H, *n* = 5–6).

To verify that this ROI approach did not substantially bias our results (given possible differences in viral diffusion rather than strictly transduction efficacy), we also analyzed the images without the ROI, using the entire slices (Figure S3). This did not substantially alter the conclusions—while the overall transduction rate is lower, due to broadening the focus to include more weakly transduced areas of a slice, the same trends were observed among the capsid variants.

In general, variants that exhibited higher rates of transduction (e.g., PHP.eB and PHP.S) tended to show higher rates of transduction across all cell types, whereas variants with lower transduction rates (AAV5, DJ) tended to show lower transduction across all cell types. Most capsid variants, including the FDA-approved AAV2 and AAV9 serotypes, showed moderate transduction, averaging 30%–50% of all cells transduced. Astrocytes tended to show higher rates of transduction than either neurons or microglia. Finally, most capsids show a negative correlation between age and transduction efficiency, indicating better transduction at younger ages (notably PHP.eB), though the variability necessitates further investigation into this finding (Figure S4).

Because future AAV screens using human brain tissue will require high-throughput methods capable of accommodating libraries of hundreds of thousands of AAV variants, we next sought to determine whether single-nucleus RNA sequencing could capture barcoded versions of these same 14 AAV variants from two patient samples (Table S1). The first sample was temporal lobe cortex from a pediatric patient with Sturge-Weber syndrome (a neurocutaneous syndrome frequently causing epilepsy), whereas the second sample was adult frontal opercular cortex overlying an insular glioma (Figure 1A). Each tissue was sectioned to 300 μm slices; six slices per patient were dosed each with a 4 μL droplet containing the combined library at a total of 2.1E9 vector genomes (each variant dosed at 2.1E9 ∗ 1/14, though the library used for the pediatric temporal lobectomy sample was missing Sch9 and therefore each remaining vector was 2.1E9 ∗ 1/13). Six control slices were dosed with a 4 μL drop of PBS. All samples again were kept in culture until 14 days after vector application, then processed for single-nucleus RNA sequencing.

**Figure 2:**
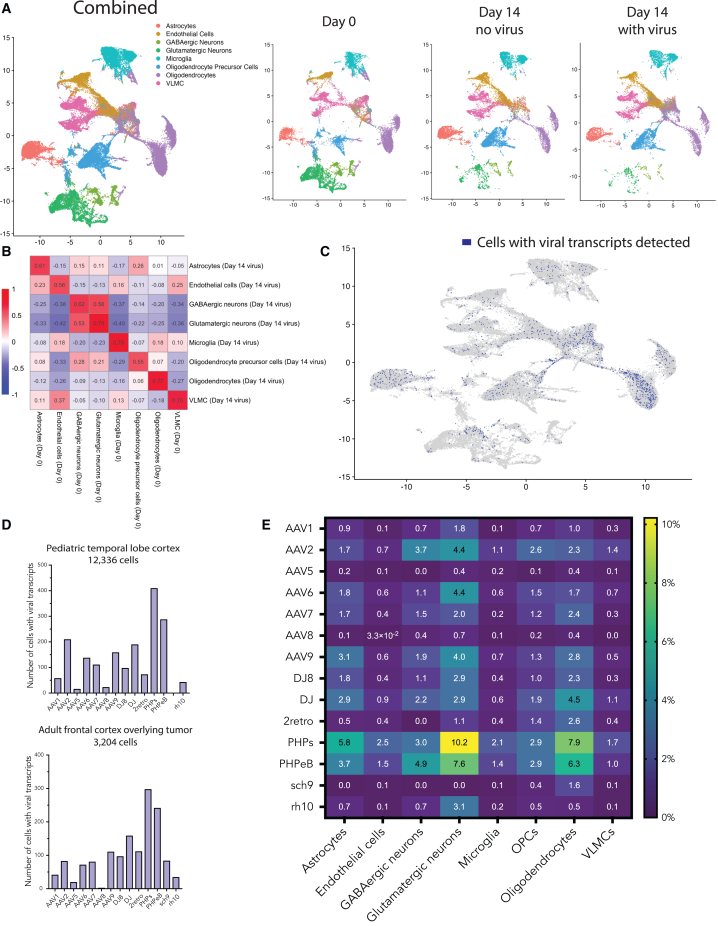
Single-nucleus RNA sequencing identifies human brain cell types transduced with AAV capsid variants (A) Samples from the pediatric temporal lobe and adult frontal lobe were sequenced and aligned independently and then combined for cluster analysis. Panels separated by day. Clusters assigned using SingleR and the Allen Institute’s middle temporal gyrus transcriptomic data. (B) Correlation matrix for log2 fold change by cell type, comparing day 0 tissue to day 14 transduced (vector added) tissue. Each cell contains the Pearson correlation coefficient. (C) UMAP of the day 14 with vector sample (15,540 cells), with dark blue cells indicating the presence of at least one viral transcript identified (1,432 cells). (D) Bar graphs showing total number of cells transduced by each AAV capsid variant for the pediatric and adult samples (pediatric sample missing Sch9). (E) Heatmap showing percentage of each cell type transduced by AAV capsid variant.

To assess whether cultured tissue remained reasonably representative of human brain cell types over the 2 weeks, we assigned clusters using SingleR with the Allen Institute’s cell types database^20^ and generated combined and subsetted UMAP plots that included all cells from day 0, day 14 with AAVs, and day 14 without AAVs. We noted similar clustering between control day 0 samples and day 14 samples both with and without vector, albeit with loss of the majority of neurons at day 14 (from 35% of total cells to 4% of total cells; Figures 2A and S5). Assessing the global transcriptional pattern of each cell type across all significantly variable genes, we found reasonable correlations between cell types' log2 fold changes for the day 0 and day 14 transduced samples (Figure 2B), indicating that the distinctive identities of each cell type were reasonably well preserved across 2 weeks in culture.

A total of 1,887 cells contained at least one viral transcript (out of 15,540 total cells) (Figure 2C). We noted good concordance of the relative transduction efficiency between the best and worst AAV variants in both the pediatric and adult single-nucleus RNA sequencing samples, as well as with our imaging data (Figure 2D compared to “all cells” [DAPI] bars in Figure 1G). Plotting separate UMAPs for each variant revealed transduction of most major cell clusters by each variant, again indicating a lack of cell-type-specific tropism for the tested AAV variants (Figure S6). We next calculated the percent transduction for each cell type by variant (Figure 2E). Given the reduced titer of each variant in the combined library (1/14^th^ of the dose used for immunofluorescence), the use of entire tissue sections for sequencing instead of focused analysis (as for imaging), sequencing depth of ∼50,000 reads per cell, and dropout effects, our absolute transduction levels seen by sequencing were expected to be significantly lower than our imaging results—however, we note that absolute levels here were of less concern than the relative transduction efficiency between variants and across cell types.

## Discussion

We found, using *ex vivo* human brain tissue, that these 14 common AAV gene therapy vectors show largely nonselective transduction across cell types, though transduction efficiency varies considerably by AAV capsid variant. PHP.eB and PHP.S showed the greatest transduction rates both by imaging and by single-nucleus RNA sequencing, whereas AAV5 was consistently among the least efficient AAVs. Notably, although PHP.eB was recognized for its broad transduction in the mouse brain (due to strain-specific Ly6a expression that facilitated blood-brain barrier crossing and likely resulted in higher effective concentrations of the vector throughout the brain^21^), PHP.S showed (in mice) minimal brain transduction apart from the brain stem, and was instead recognized for efficient targeting of sensory neurons, indicating species-specific tropism differences when compared with our human data here.^22^ The two variants in clinical use, AAV2 and AAV9, showed moderate transduction by imaging and single-nucleus RNA sequencing. Further, Sch9 was found in mice to selectively label subventricular zone neurons; in human brain tissue, Sch9 shows moderate transduction of many cell types. In comparison to non-human primate models, AAV6 for example has been shown to be a neurotropic variant in prior NHP work (with few astrocytes), whereas in our human tissue sections it shows by immunostaining a slightly greater astrocytic than neuronal transduction rate (San Sebastian et al., *Gene. Ther.*, 2013).^23^ In the search for cell-type specific vectors, we think that the use of human tissue will be a critical adjunct for defining the tropism of vectors, given the known variability in cell-surface molecules between species. For intracranial therapies in which cell-type specificity is paramount--due to concerns for safety or efficacy--it will be important to test vectors using the cell types we seek to target, rather than those of a different species.

Transgene protein expression (GFP) was generally most prominent in astrocytes, though transgene mRNA seems more evenly distributed. In order to promote strong protein expression for improved gene therapies, the balance of factors that control transgene expression in human brain cell types will need to be a focus of future study.^24^^,^^25^^,^^26^ We note that the nonselective transduction of these AAV capsid variants may be due to their initial popularization in mouse studies. That is, the variants may share features that helped them show promise in mouse and gain widespread use; in most cases, this meant they were selected for broad CNS transduction. Moreover, several are closely related.

We have used only a single promoter, CAG, one of the most common in neurological clinical trials, as well as in pre-clinical animal work. The rationale for this is that when assessing capsids for their cell type specificity, one should use the broadest and strongest promoter (and in this case one of the most clinically relevant promoters); we wanted to examine the cell type specificity of the capsids in this study, not a combination of the capsid and regulatory elements. However, it will certainly be the case that various enhancers, in combination with specific AAV capsid variants, will help narrow cell-type-specific expression. Enhancer identification and expression analysis in human brain tissue is therefore the focus of ongoing work. It is notable that there is a substantial loss of neurons over 2 weeks in culture. This may be due to their susceptibility to hypoxia during tissue collection and transport or the use of defined culture media, which is not a perfect substitute for *in vivo* conditions. Some electrophysiology groups have had better success characterizing and culturing neurons using human CSF as a culture media, which is an avenue for future AAV transduction studies using *ex vivo* human tissue.^16^ Near-normal pediatric tissue could plausibly be more robust than adult, as seen in our data, though this requires substantiation with additional samples. Given the loss described above, it is notable that the correlation in gene expression profile of the neurons that remain is relatively strong (0.62, 0.79), and that transduction at the RNA level is higher in neurons than many cell types (Figure 2E), despite relatively poorer transduction as assessed by immunostaining (Figure 1H). Whether this is due to translation, protein stability, or some other factor is a topic of ongoing study.

The lack of pial or ependymal layers covering our slices means that this method most closely approximates direct intraparenchymal injections rather than systemic or intraventricular delivery and should be interpreted accordingly.^27^ Our findings indicate that intraparenchymal gene therapy injections with these capsids can be expected, when carrying the CAG promoter, to transduce most cell types present, with notably strong protein expression in astrocytes.

Finally, human patient samples are reasonably expected to be more heterogeneous than laboratory strains of inbred mice or cell culture lines, and so more variability should be expected, which may be considered a limitation. As others have argued, however, testing therapies in these more variable but relevant conditions should help identify interventions whose signal is able to surmount such variability and therefore predict clinical trial performance more reliably.^14^^,^^28^ Together, these findings underscore the continued need to develop cell-type-specific AAV variants and the importance of using the ultimate intended target of such vectors, human brain tissue, for precise tropism characterization.^9^^,^^29^

## Materials and methods

### Human brain tissue samples

Patients planned to undergo resective neurosurgical procedures at three major neighboring academic hospitals were approached and consented pre-operatively by a member of the study team (Baylor College of Medicine, IRB H-51865) for any specimens not needed for pathologic diagnosis. In the case of pediatric patients or adults unable to consent, parents or medical decision-makers were consented.

Specimens were collected from pathology following tissue sectioning and gross examination by board-certified neuropathologists, or directly from the operating room in sterile plastic specimen containers, usually within minutes and no longer than 45 min after resection, into which we poured ice-cold, pre-carbogenated (>20 min bubbling time, 95%/5% O2/CO2) NMDG-aCSF, following the protocol from Park et al.^14^ Tissue was rapidly transported to the lab on ice, where it was manually sectioned with scalpels into pieces ∼1 cm × 1 cm. Tissue pieces were placed into a retractable tube, and molten ultra-low melting agarose (Sigma A2576) was poured over top. Using a cold clamp, the agarose was rapidly solidified, at which point the agarose cylinder was removed and superglued onto a tape-covered vibratome mount. Tissues were oriented so that the sectioning would be done perpendicular to the cortical surface, parallel to neuronal projections. Tissues were sectioned to 300 μm on a Leica VT1200 vibratome in cold aCSF that was being continuously bubbled with carbogen. We then moved the slices to room temperature aCSF that was continuously bubbled with carbogen for 15–30 min, after which the slices were plated individually onto membranes inserts (CellTreat #230617) overlying 600 μL of organotypic slice culture media (OSCM) as detailed in Park et al., 2022. Any excess media or aCSF was aspirated off the membrane surface, and the plates were kept in a humidified tissue culture incubator at 37°C, 95% humidity, and 5% CO2. The tissue underwent daily media changes (400 μL removed and added to the surrounding well, avoiding any contact with the tissue slice).

### AAV production

All AAVs were packaged in-house by the Texas Children’s Hospital Jan and Dan Duncan Neurological Research Institute’s Neuroconnectivity Core (supported via NIH P50HD103555). Viral capsid gene sequences are found in Table S1. The CAG-EGFP-WPRE-hGH polyA signal was modified by removing the bGH polyA signal from Addgene #37825 (gift from Ed Boyden) and replacing it with the hGH polyA signal. The entire plasmid sequence is found in Table S1.

#### Cell culture and transfection

A three-vector system was used for AAV production (Cell Biolabs). HEK-293 cells were plated in 15 cm dishes at a density that yielded ∼70% confluency the following day. Cells were then transfected in each plate with 25 μg helper plasmid, 25 μg serotype-specific AAV vector, and 25 μg of AAV shuttle vector using polyethylenimine (PEI). In a 1:3 ratio (μg DNA:μg PEI), the solution was added dropwise to cells. After 4–6 h, the medium was changed to DMEM, 5% FBS, 1x Penicillin/Streptomycin. Forty-eight to seventy-two hours later, transfected cells were harvested using a cell scraper. Cells were pelleted by centrifugation at 3,500 rpm for 10 min at 4°C. The supernatant was removed, and the pellet resuspended in TMN (50 mM Tris pH 8.0, 5 mM MgCl_2_, 0.15M NaCl) at a concentration of 1mL/plate. The resuspended cells were frozen at −80°C overnight.

#### Purification

Ten microliters of DNaseI (10 mg/mL) and 10 μL RNase A (1 mg/mL) were added to each plate of defrosted cells in TMN. Plates were incubated at 37°C for 30 min, shaking frequently. One hundred microliters of 5% sodium deoxycholate solution in water was added to each plate and mixed gently. Plates were then incubated at 37°C for 10 min. The suspended samples were transferred from the plates to tubes and placed on ice for 15 min. The tubes were then centrifuged at 3,700 rpm for 10 min, and the supernatant was collected.

#### Iodixanol gradient

OptiPrep (Millipore Sigma D1556-250ML), or iodixanol, was purchased as a 60% (W/V) stock in water; 15%, 25%, and 40% dilutions of iodixanol were made in PBS-MK (1x PBS, 1 mM MgCl_2_, 2.5 mM KCl); 2.5 μL phenol red solution (0.5% stock in PBS-MK) was added per 1 mL of iodixanol solution in the 25% and 60% fractions. The gradient was loaded to the bottom of Beckman OptiSeal 16 × 67mm tubes (Cat# 362181), starting with 1.5 mL 15% iodixanol, 1.3 mL 25%, 1.4mL 40%, and finally 1.3mL 60% iodixanol. The supernatant collected from the previous purification step was then placed on top of the gradient. Tubes were centrifuged at 60,000 rpm for 90 min in a Beckman NVT 65 rotor. The clear band below the 60% mark (and below the white cellular debris layer) was collected using a needle and syringe. The collected volume from each tube was approximately 1.5 mL.

#### Concentration

The goal of this step was to remove the OptiPrep and concentrate the AAV using an Amicon Ultra-15 Centrifugal Filter (Millipore Sigma, UFC9 100 24). An Amicon column was equilibrated with 15 mL of DPBS (no Mg, no Ca) by centrifugation at 2,500 rpm for 5 min. The collected band from the OptiPrep gradient was mixed with approximately 40 mL of DPBS. The samples were run in batches through the Amicon filter, discarding the filtrate between spins. The viral vector was then washed three times with 15 mL of DPBS with centrifugation after each wash at 2,500 rpm for 10 min. The viral vector was collected to a sterile microcentrifuge tube, aliquoted, and frozen to −80°C.

#### Viral vector titer

Titering of virus was performed using Applied Biological Materials qPCR AAV Titer Kit (Cat# G931) and following the manufacturer’s recommended protocol. The ABM qPCR AAV Titer Kit uses qPCR with primers targeting the 3′ ITR, which in our case is identical across all capsids used here. (The cargo plasmid remains identical apart from unique custom 25 bp barcodes that are different for each capsid.) Viral preparations were first diluted to ∼10^8^ GC/mL before undergoing viral lysis at room temperature for 3 min. A standard curve was generated using five 10-fold serial dilutions of provided Standard Control DNA (dilutions 1/100 to 1/100,000). qPCR components and cycling conditions are found within the manufacturer’s accompanying product datasheet. Final titer analysis was performed using the manufacturer’s provided calculation file.

When combined into the library, vectors were packaged separately and titers generated independently; based on these titers, they were then combined in equimolar amounts.

### Viral vector application

On the day after collection (post-operative day 1), 2.1E9 vector is applied (in a volume of 4 μL where sterile PBS is used to supplement volume up to 4 μL) to the surface of the slice. The 4 μL droplet at the end of a 10 μL pipette tip is touched to the center of the slice. The media underlying the slices underwent daily changes until 14 days after vector addition, at which time they were either flash frozen in liquid nitrogen (for later single-nucleus isolation and sequencing) or fixed in 4% PFA in PBS (for imaging). Control slices had 4 μL of PBS pipetted instead of AAVs. For imaging, one variant per well was used, at a dose of 2.1E9. For the combined pool for sequencing, the total dose was 2.1E9, such that each variant was present at 1/14 ∗ 2.1E9 for the adult sample and 1/13 ∗ 2.1E9 for the pediatric sample (which was missing Sch9).

### Histology, imaging, and analysis

Fourteen days after vector addition, tissues were fixed in 4% PFA overnight at 4°C by removing media from the wells and adding 4% PFA; the next day, PFA was removed and replaced with 20% sucrose overnight and then 30% sucrose overnight for cryopreservation. Tissues were then mounted exposed surface down in OCT such that the first 20 μm cryostat slices would be the surface on which vector was applied. Sections were affixed to typically four slides sequentially, such that each slide contained some superficial, middle, and deeper layers of each tissue slice. Slides were stored in −20°C. Slides were rinsed with PBS-0.1% Triton X for 10 min, then blocked overnight at 4°C in PBS-0.1%T+10% normal donkey serum. Slides were then placed tissue-side down in thin slide folders (Amazon OakRidge Products B00X6L1NM4) over 600 μL of combined 1:1,000 chicken anti-GFP (Abcam 13970), rabbit anti-NeuN (Abcam 177487), and mouse anti-GFAP (Abcam 279290) or 1:500 rabbit anti-Iba1 (Thermo Fisher PA5-27436) overnight at 4°C. Slides were washed five times for at least 5 min each time in PBS-0.1%T and similarly placed on 600 μL secondary antibody (1:1,000 goat anti-chicken Alexa 488, goat anti-rabbit Alexa 546, and goat anti-mouse Alexa 633). Slides were again washed 5x for at least 5 min each, partially dried, covered with fluoromount G with DAPI, and coverslipped.

Approximately 4–6 sections were present per slide depending on section size. All sections were inspected visually under 10X GFP epifluorescence to confirm similarity, and the two sections with highest GFP intensity were selected for complete confocal imaging on a Leica SP8 microscope using a 10X objective/NA 0.4. Because we care most about approximating, as best as possible, the percentage of cell-type transduction at the tip of a catheter used for intraparenchymal injection, we wanted to analyze the best possible transduction capabilities of each vector. We also needed to control for variability in slice size and eccentric transduction due to liquid slide/spread of the pipette drop of vector. In order to mitigate these factors, a consistent 2 mm circular ROI was applied to every section and manually centered over the area of most dense GFP expression. (Example images are found in Figure S1A with overlying ROIs that were used.) In this way, larger slices, but with a similar area of transduction, would not be penalized simply for being larger and having more untransduced (or more weakly transduced) areas—the brightest area of transduction on each slice would be analyzed similarly. This relies on the assumption that the area of brightest GFP signal is the area that saw the greatest amount of vector, which, while not certain, is a reasonable assumption.

Using a custom FIJI/ImageJ program (https://github.com/jpmcginnis/HumanAAVProject/blob/main/imageJ-imagesorterforlifloop), .lif files were converted to .tif files, and the .tif files were automatically scanned by a standardized 2 mm circle to find the location that would maximize GFP signal within the circle (https://github.com/jpmcginnis/HumanAAVProject/blob/main/imageJ-automatedROIloopandclose). The program saved a screenshot of where that circle was placed and then saved separately an image only of the data within the 2 mm circle. Using that restricted image, ROIs were drawn around each DAPI nucleus using another custom ImageJ/FIJI program, and the average intensity across each individual ROI was calculated separately for each of the four channels. (Code: https://github.com/jpmcginnis/HumanAAVProject/blob/main/imageJ-Macro2-DAPIchannel1-loop.).

We additionally carried out an alternative analysis without the 2 mm ROI, in which case the analysis was the same as above except that the entire imaged slice was used.

Thresholds for positive/negative GFP cells were assigned by identifying the maximally brightest ROI of the untransduced control slices for each separate patient sample and setting the threshold at that value for that patient’s imaging analysis (such that any ROI brighter than the maximally bright “untransduced” ROI would be called GFP^+^); the threshold was different for different patient samples (https://github.com/jpmcginnis/HumanAAVProject/blob/main/googlecolab-Step1-Ch1DAPICh2-633-Ch3-488-Ch4-546-generatepercentpositiveneedtosetthresholds). Thresholds for NeuN^+^, GFAP^+^, and Iba1^+^ were set by examining representative images’ ROIs within that channel and selecting a threshold that would include clearly positive cells but disregard negative cells; this was typically around the mean value for the channel. Using custom Python language in Google Colabs, the ROIs were then identified as either positive or negative for GFP or the other channels, and the percentage of a certain channel (DAPI, anti-rabbit 546, or anti-mouse 633) positive for GFP was calculated. In order for an image to be included, it needed to have at least 30 cells of that type to prevent conclusions being made from low numbers of cells. Graphs were created using Graphpad Prism 10. All code available at https://github.com/jpmcginnis/HumanAAVProject/tree/main.

### Single-nucleus RNA sequencing

For the tissue sections that received barcoded viral libraries (total titer 2.1E9 in 4 μL PBS per tissue slice), 14 days after vector application the tissue was gently removed from its well, flash frozen in liquid nitrogen, and stored at −80°C. On the day of processing, frozen tissue was dissociated using GentleMACS nuclei isolation protocol (nuclei isolation buffer [Miltenyi Biotec, cat# 130-128-024], Protector RNAse Inhibitor [Millipore Sigma, cat# 3335402001], GentleMACS C tubes [Miltenyi Biotec, cat# 130-093-237], GentleMACS Octo Dissociator [Miltenyi Biotec, cat# 130-096-427], MACS SmartStrainers 70 μm [Miltenyi Biotec, cat# 130-098-462], MACS SmartStrainers 30 μm [Miltenyi Biotec, cat# 130-098-458]). In brief, samples were placed in 2 mL of Miltenyi Nuclei Isolation Buffer and Protector RNAse Inhibitor in GentleMACS C tubes. Samples then underwent the preprogrammed “nuclei isolation” program on a GentleMACS Octo Dissociator. Immediately after dissociation, samples were strained through a 70 μm MACS SmartStrainer and collected in a 15 mL tube, centrifuged at 300xg for 5 min at 4°C. The supernatant was extracted and discarded and the resulting pellet resuspended in 1 mL of ice-cold PBS. Resuspended samples were then run through a 30 μm MACS SmartStrainer, centrifuged, and resuspended in ∼250–500 μL. Nuclei were then FACS sorted using DAPI on a Sony MA900 in the Baylor Cytometry and Cell Sorting Core.

#### Library preparation

The single-cell gene expression libraries were prepared according to the Chromium Single Cell Gene Expression 3′v3.1 instruction (PN-1000121, PN-1000120, PN-1000213, 10x Genomics). Briefly, single cells, reverse transcription (RT) reagents, Gel Beads containing barcoded oligonucleotides, and oil were loaded on a Chromium controller (10x Genomics) to generate single-cell GEMS (Gel Beads-In-Emulsions) where full-length cDNA was synthesized and barcoded for each molecule of mRNA (UMI) and each single cell (cell barcode). Subsequently, the GEMS were broken, and cDNA from each single cell was pooled. Following cleanup using Dynabeads MyOne Silane Beads, cDNA is amplified by PCR. The amplified product was fragmented to optimal size before end-repair, A-tailing, and adaptor ligation. The final library was generated by amplification.

#### Cell-type annotation and barcode identification

Fastq files were uploaded to the 10X Cloud Analysis web browser. A custom reference genome was created using human GRCh38 to which the fourteen 25 base pair barcode sequences were added (barcode sequences found in Table S1); this was used to align the fastq files. R/Seurat: each sample’s (day 0, day 14 without vector, day 14 with vector) filtered matrix HD5 file was downloaded from the 10X cloud and was used to generate a Seurat object. These Seurat objects were then integrated to create a combined object for joint UMAP creation. The Allen Institute Brain Atlas transcriptomics explorer cell-type CSV for human middle temporal gyrus (Gene expression by cell type, medians) was downloaded, and SingleR was used to assign cell types. These were then collapsed into general categories (Astrocyte, Glutamatergic neuron, Oligodendrocyte, etc.), and the FindMarkers() function in Seurat was used to identify differentially expressed genes between each cluster for a given sample by day (separately for day 0, day 14 without vector, and day 14 with vector). We generated a list of all genes showing differential expression between any two clusters for that given sample, log2fold changes, and *p* values, which was downloaded for each cell type, and then Pearson correlation was calculated across all differentially expressed genes between pairs of cell types; genes not present in one of the two cell types being compared were discarded (code at https://github.com/jpmcginnis/HumanAAVProject/tree/main). Graphs and UMAPs were created in Graphpad Prism 10 or using ggplot2.

#### Statistical analysis

Given the lack of normality, imaging data were analyzed by nonparametric Kruskal-Wallis tests with Dunn post-hoc tests, adjusted for multiple comparisons where appropriate. *n* = 3 to 8, depending on the AAV variant and cell type because not all cell types were sufficiently represented (we required at least 30 of that cell type to be present to analyze that cell type for that tissue/variant). All statistical tests were two-sided with α = 0.05. Data are presented as mean ± SEM unless otherwise indicated. For single-nucleus RNA sequencing analysis, differential expression analysis was performed using Seurat/FindMarkers() using the nonparametic Wilcoxon rank-sum test with Benjamini-Hochberg correction for multiple comparisons.

## Data availability

All code is currently available at https://github.com/jpmcginnis/HumanAAVProject, and images are pending upload at Biostudies (ebi.ac.uk). Fastq or Seurat objects are immediately available on request and are being uploaded to GEO.

## Acknowledgments

We thank the hundreds of patients who have generously participated in this project, as well as Ying, Rong, Hilary, Gemma, Megan, Jennifer, Loretta, Glenn, Omar, Waseem, Johannah, Neal, Juan, Melanie, Mira, the Arenkiel lab, cytometry core, and the unnamed operating room, pathology, and research staff who have contributed so much time and effort to this work.

The majority of the funding for this project came from a generous gift from the Wilsey family. The project was also supported in part by the 10.13039/100012050NRI Neuroconnectivity and Viral Core (supported via 10.13039/100000002NIH P50HD103555) and the RNA In Situ Hybridization Core facility at Baylor College of Medicine (supported by a Shared Instrumentation grant from the 10.13039/100000002NIH 1S10OD016167 and the NIH IDDRC grant P50 HD103555 from the Eunice Kennedy Shriver National Institute Of Child Health & Human Development). This project was further supported by the Cytometry and Cell Sorting Core at Baylor College of Medicine with funding from the 10.13039/100004917CPRIT Core Facility Support Award (CPRIT-RP180672), the 10.13039/100000002NIH (CA125123 and RR024574) and the assistance of Joel M. Sederstrom, and by the Single Cell Genomics Core at Baylor College of Medicine with funding from the 10.13039/100004917CPRIT RP200504 and the 10.13039/100000002NIH S10OD032189.

## Author contributions

J.P.M., J.O.G., D.J.C., and B.A. designed the project. J.P.M. and D.J.C. obtained IRB approval. Human brain tissue and acquisition was carried out by J.P.M., J.O.G., C.G., S.M., B.B., S.S., E.J., M.B., C.M., G.A., A.P., A.J., S.S., H.W., S.G., G.R., and D.J.C. Vector production was performed by Y.W., Z.C., and J.O.G. Tissue processing and vector application was done by J.P.M., J.O.G., C.G., S.M., S.B., A.A., E.T., M.C.L., and S.C. J.P.M., J.L., C.G., S.M., E.J., and S.C. performed the imaging and carried out the analysis. Single-nucleus library prep was done by J.P.M., C.G., S.M., and S.B. Single-nucleus RNA sequencing analysis was done by J.P.M., S.M., A.S.H., C.G., and S.B. B.A., D.J.C., and J.O.G. obtained the funding for the project. Initial manuscript draft was written by J.P.M., with manuscript editing and review done by all authors.

## Declaration of interests

The authors declare no conflict of interest with the study or any of the topics discussed.

## Supplemental information

Document S1. Figures S1–S6 and Table S1

Table S2. Estimated AAV dosing per mg of brain tissue in intraparenchymal, convection-enhanced delivery trials For clinical trials that have published their vector doses and volume infused, we assume the 1:1 to 1:3 infusate volume to brain volume infused that has been reported, in order to estimate the number of viruses per mg of brain tissue.

Document S2. Article plus supplemental information
