# Supplementary material for: Common AAV gene therapy vectors show nonselective transduction of ex vivo human brain tissue
Source: Mol Ther Methods Clin Dev. 2025 May 21;33(2):101494. doi: 10.1016/j.omtm.2025.101494 (PMC12169722; doi:10.1016/j.omtm.2025.101494)
Supplement: Document S1. Figures S1–S6 and Table S1 [file mmc1.pdf]

OMTM, Volume *33*

Supplemental information

Common AAV gene therapy vectors show nonselective transduction of *ex vivo* human brain tissue

JP McGinnis, Joshua Ortiz-Guzman, Maria Camila Guevara, Sai Mallannagari, Benjamin D.W. Belfort, Suyang Bao, Snigdha Srivastava, Maria Morkas, Emily Ji, Angela Addison, Evelyne K. Tantry, Sarah Chen, Ying Wang, Zihong Chen, Kalman A. Katlowitz, Jeffrey J. Lange, Melissa M. Blessing, Carrie A. Mohila, M. Cecilia Ljungberg, Guillermo Aldave, Ali Jalali, Akash Patel, Sameer A. Sheth, Howard L. Weiner, Shankar Gopinath, Ganesh Rao, Akdes Serin Harmanci, Daniel J. Curry, and Benjamin R. Arenkiel

Table S1. List of capsids and their sequences, and barcodes used for pooled libraries

Capsid	Sequence
AAV1	atggctgccgatggttatcttccagattggctcgaggacaacctctctgagggcattcgcgagtggtgggacttgaaacctggagccccgaagccca<br>aagccaaccagcaaaagcaggacgacggccggggtctggtgcttcctggctacaagtacctcggacccttcaacggactcgacaagggggagcc<br>cgtcaacgcggcggacgcagcggccctcgagcacgacaaggcctacgaccagcagctcaaagcgggtgacaatccgtacctgcggtataacca<br>cgccgacgccgagtttcaggagcgtctgcaagaagatacgtcttttgggggcaacctcgggcgagcagtcttccaggccaagaagcgggttctcga<br>acctctcggtctggttgaggaaggcgctaagacggctcctggaaagaaacgtccggtagagcagtcgccacaagagccagactcctcctcgggca<br>tcggcaagacaggccagcagcccgctaaaaagagactcaattttggtcagactggcgactcagagtcagtccccgatccacaacctctcggagaa<br>cctccagcaacccccgctgctgtgggacctactacaatggcttcaggcggtggcgcaccaatggcagacaataacgaaggcgccgacggagtgg<br>gtaatgcctcaggaaattggcattgcgattccacatggctgggcgacagagtcatcaccaccagcacccgcacctgggccttgcccacctacaata<br>accacctctacaagcaaatctccagtgcttcaacgggggccagcaacgacaaccactacttcggctacagcacccccctgggggtattttgatttca<br>acagattccactgccacttttcaccacgtgactggcagcgactcatcaacaacaattggggattccggcccaagagactcaacttcaaactcttca<br>acatccaagtcaaggaggtcacgacgaatgatggcgtcacaaccatcgctaataaccttaccagcacggttcaagtcttctcggactcggagtacc<br>agcttccgtacgtcctcggctctgcgcaccagggctgcctccctccgttcccggcggacgtgttcatgattccgcaatacggctacctgacgctcaa<br>caatggcagccaagccgtgggacgttcatccttttactgcctggaatatttcccttctcagatgctgagaacgggcaacaactttaccttcagctacac<br>ctttgaggaagtgcctttccacagcagctacgcgcacagccagagcctggaccggctgatgaatcctctcatcgaccaatacctgtattacctgaac<br>agaactcaaaatcagtccggaagtgcccaaaacaaggacttgctgtttagccgtgggtctccagctggcatgtctgttcagcccaaaaactggctac<br>ctggaccctgttatcggcagcagcgcgtttctaaaacaaaaacagacaacaacaacagcaattttacctggactggtgcttcaaaatataacctca<br>atgggcgtgaatccatcatcaaccctggcactgctatggcctcacacaaagacgacgaagacaagttctttcccatgagcggtgtcatgatttttgga<br>aaagagagcgccggagcttcaaacactgcattggacaatgtcatgattacagacgaagaggaaattaaagccactaaccctgtggccaccgaaag<br>atttgggaccgtggcagtcaatttccagagcagcagcacagaccctgcgaccggagatgtgcatgctatgggagcattacctggcatggtgtggcaa<br>gatagagacgtgtacctgcagggtcccatttgggccaaaattcctcacacagatggacactttcacccgtctcctcttatgggcggctttggactcaag<br>aacccgcctcctcagatcctcatcaaaaacacgcctgttcctgcgaatcctccggcggagttttcagctacaaagtttgcttcattcatcacccaata<br>ctccacaggacaagtgagtgtggaaattgaatgggagctgcagaaagaaaacagcaagcgctggaatcccgaagtgcagtacacatccaattatg<br>caaaatctgccaacgttgattttactgtggacaacaatggactttatactgagcctcgccccattggcacccgttaccttacccgtcccctgtaa
AAV2	atggctgccgatggttatcttccagattggctcgaggacactctctctgaaggaataagacagtggtggaagctcaaacctggcccaccaccaccaa<br>agcccgcagagcggcataaggacgacagcaggggtcttgtgcttcctgggtacaagtacctcggacccttcaacggactcgacaagggagagccg<br>gtcaacgaggcagacgccgcggccctcgagcacgacaaagcctacgaccggcagctcgacagcggagacaacccgtacctcaagtacaacca<br>cgccgacgcggagtttcaggagcgccttaaagaagatacgtcttttgggggcaacctcggacgagcagtcttccaggcgaaaaagagggttcttgaa<br>cctctgggcctggttgaggaacctgttaagacggctccgggaaaaaagaggccggtagagcactctcctgtggagccagactcctcctcgggaacc<br>ggaaaggcgggccagcagcctgcaagaaaaagattgaattttggtcagactggagacgcagactcagtacctgacccccagcctctcggacagcc<br>accagcagccccctctggtctgggaactaatacgatggctacaggcagtggcgcaccaatggcagacaataacgagggcgccgacggagtgggt

	aattcctcgggaaattggcattgcgattccacatggatgggcgacagagtcatcaccaccagcacccgaacctgggccctgcccacctacaacaa<br>ccacctctacaaacaaatttccagccaatcaggagcctcgaacgacaatcactactttggctacagcaccccttgggggtattttgacttcaacaga<br>ttccactgccacttttcaccacgtgactggcaaagactcatcaacaacaactggggattccgacccaagagactcaacttcaagctctttaacattc<br>aagtcaaagaggtcacgcagaatgacggtacgacgacgattgccaataaccttaccagcacggttcaggtgtttactgactcggagtaccagctcc<br>cgtacgtcctcggctcggcgcatcaaggatgcctcccgccgttcccagcagacgtcttcatggtgccacagtatggatacctcaccctgaacaacg<br>ggagtcaggcagtaggacgctcttcattttactgcctggagtactttccttctcagatgctgcgtaccggaaacaactttaccttcagctacacttttgag<br>gacgttcctttccacagcagctacgctcacagccagagtctggaccgtctcatgaatcctctcatcgaccagtacctgtattacttgagcagaacaa<br>acactccaagtggaaccaccacgcagtcaaggcttcagttttctcaggccggagcgagtgacattcgggaccagtctaggaactggcttcctggac<br>cctgttaccgccagcagcgagtatcaaagacatctgcggataacaacaacagtgaatactcgtggactggagctaccaagtaccacctcaatggc<br>agagactctctggtgaatccgggcccggccatggcaagccacaaggacgatgaagaaaagtttttcctcagagcggggttctcatctttgggaagc<br>aaggctcagagaaaacaaatgtggacattgaaaaggtcatgattacagacgaagaggaaatcaggacaaccaatcccgtggctacggagcagtat<br>ggttctgtatctaccaacctccagagaggcaacagacaagcagctaccgcagatgtcaacacacaaggcgttcttccaggcatggtctggcagga<br>cagagatgtgtaccttcaggggcccatctgggcaaagattccacacacggacggacattttcacccctctcccctcatgggtggattcggacttaaa<br>caccctcctccacagattctcatcaagaacaccccggtacctgcgaatccttcgaccaccttcagtgcggcaaagtttgcttccttcatcacacagt<br>actccacgggacaggtcagcgtggagatcgagtgggagctgcagaaggaaaacagcaaacgctggaatcccgaaattcagtacacttccaacta<br>caacaagtctgttaatgtggactttactgtggacactaatggcgtgtattcagagcctcgccccattggcaccagatacctgactcgtaatctgtaa
AAV5	ttaaaggggtcgggtaaggtatcgggttccgataggtctggtggttctgtattccccggtgctgtccggggcaaagtccacaaactgggggtcgttgtag<br>ttgtttgtgtactggatctctgggttccacctcttggagttttccttcttgagctcccactccatctccacggtgacctgcccggtgctgtactgggtgatga<br>agctgctgacgggcacgtccgagaagctggtgatatttccgggcacaggcgtgttcttgatgagcatcatgggcggtgggtgtttgagtccgaatccgc<br>ccatggccggagaggggtgaaagtgcgcccccgtctctgggatcttggcccagatgggtccttggaggtacacgtccctctccatccacacgctgcc<br>gggcacgatttcctggaggttgtacgtgccggtcgcgggggcagtggtggagctctggttgttggtggccatctgcccgccgacgttgtacgccacgcg<br>gttcaccggctgcgtctcgctctcgctggtgatgagcatgttgccctcgaggtacgtggcggtggtgcccgggttcgccggctggctgttgaagatcata<br>gtgttctccagggcataggtgttgctgccctggaggttgttggtcatgccgttcggctgcgggggcacctggtaactcgcgccctcgagctccatcctat<br>tggtcgtggcgaaggcgctgacactggcgcggttgaccccggagcccaggttccagccctgggttcggcccatgggccccgggaaccagtttttgta<br>ggtgttggcgtatctcccggccaggttcttgttgaactggactccgccagtgttatttgtgctcacgaagcggtacaagtactggtccaccagcgggttg<br>gccagcttgaacaggttctgactgggagcgaagctggagtggaagggcacctcctcaaagttgtaggtaaactcaaagttgttgcccgttctcagcat<br>cttgctgggaaagtactctaggcagaagaagctgctcctctcggtgggattttctgtgttgtcgcggttcagcgtcgcgtaaccgtactgcggcagcgta<br>aagacctgcggagggaaggccggcaggcatccctcggtcccgttgccgacgacgtagggcagctggtagtcgtcgtccgtaaacacttggacggtg<br>gaggtgaggttgttggcgatggtggtggtggagtcctgcaccgtgacctctttgacttgaatgttgaagattttgactctgagggaccggggtctgaagcc<br>ccagtagttgttgatgagtctttgccagtctcgggggctccagtggctgtggaagcggttaaagtcaaagtacccccagggggtgctgtatccaaagta<br>ggcgttggcgttgcttccgtcgacggagccgcttttgatctctcggtactggtggttgttgtagctgggcagcacccaggttcgggtggacttggtgacga

	ctctgtcccccatccacgtggaatcgcaatgccaatctcccgaggcattgcccactccatcggcaccttggttattgtcgcccaatgggccgccacc<br>tcccgcagacattgtatcagctcccaaacttgaggctggttgggctgggatttgcagctgctgggatccgctgggtccagcttcggcgtctgacgaggt<br>ggaaggcttggagtcctcttcggtccgagccttctttcttttggaaagtggtcgtctatccgctttccggtaggggccgtcttagcaccctcttcaacca<br>ggccaaaaggttcgagaacccttttcttggcctgaaagactgcctttccgaggtttcccccgaaggatgtgtcgtcggcgagcttctcctgaaactcgg<br>cgtccgcgtggttgtacttgaggtaggggttgtctcccgcctcaagctgctcgttgtacgagatgtcgtgctctcgcgcgacctcgtctgccctgttgaca<br>ggctctcctcgatcgagaccgtttccgggtccgagatagttataaccaggcagcacaagaccacgggcttgatcttgatgctgctgattgggttttggtt<br>tcggtgggcccgcttcaaggcccaaaaactcgcgaagaccttcaccaacttcttccaaccaatctggagggtgatcaacaaaagacat
AAV6	atggctgccgatggttatcttccagattggctcgaggacaacctctctgagggcattcgcgagtggtgggacttgaaacctggagccccgaaaccca<br>aagccaaccagcaaaagcaggacgacggccggggtctggtgcttcctggctacaagtacctcggacccttcaacggactcgacaagggggagcc<br>cgtcaacgcggcggatgcagcggccctcgagcacgacaaggcctacgaccagcagctcaaagcgggtgacaatccgtacctgcggtataacca<br>cgccgacgccgagtttcaggagcgtctgcaagaagatacgtcttttgggggcaacctcgggcgagcagtcttccaggccaagaagagggttctcga<br>accttttggtctggttgaggaaggtgctaagacggctcctggaaagaaacgtccggtagagcagtcgccacaagagccagactcctcctcgggcatt<br>ggcaagacaggccagcagcccgctaaaaagagactcaattttggtcagactggcgactcagagtcagtccccgacccacaacctctcggagaac<br>ctccagcaacccccgctgctgtgggacctactacaatggcttcaggcggtggcgcaccaatggcagacaataacgaaggcgccgacggagtgggt<br>aatgcctcaggaaattggcattgcgattccacatggctgggcgacagagtcatcaccaccagcacccgaacatgggccttgcccacctataacaa<br>ccacctctacaagcaaatctccagtgcttcaacgggggccagcaacgacaaccactacttcggctacagcacccccctgggggtattttgatttcaa<br>cagattccactgccatttctcaccacgtgactggcagcgactcatcaacaacaattggggattccggcccaagagactcaacttcaagctcttcaac<br>atccaagtcaaggaggtcacgacgaatgatggcgtcacgaccatcgctaataaccttaccagcacggttcaagtcttctcggactcggagtaccagt<br>tgccgtacgtcctcggctctgcgcaccagggctgcctccctccgttcccggcggacgtgttcatgattccgcagtacggctacctaacgctcaacaa<br>tggcagccaggcagtgggacggtcatccttttactgcctggaatatttcccatcgcagatgctgagaacgggcaataactttaccttcagctacacctt<br>cgaggacgtgcctttccacagcagctacgcgcacagccagagcctggaccggctgatgaatcctctcatcgaccagtacctgtattacctgaacag<br>aactcagaatcagtccggaagtgcccaaaacaaggacttgctgtttagccgggggtctccagctggcatgtctgttcagcccaaaaactggctacct<br>ggaccctgttaccggcagcagcgcgtttctaaaacaaaaacagacaacaacaacagcaactttacctggactggtgcttcaaaatataaccttaat<br>gggcgtgaatctataatcaaccctggcactgctatggcctcacacaaagacgacaaagacaagttctttcccatgagcggtgtcatgatttttggaaa<br>ggagagcgccggagcttcaaacactgcattggacaatgtcatgatcacagacgaagaggaaatcaaagccactaaccccgtggccaccgaaaga<br>tttgggactgtggcagtcaatctccagagcagcagcacagaccctgcgaccggagatgtgcatgttatgggagccttacctggaatggtgtggcaaga<br>cagagacgtatacctgcagggtcctatttggccaaaattcctcacacggatggacactttcacccgtctcctctcatgggcggctttggacttaagc<br>acccgcctcctcagatcctcatcaaaaacacgcctgttcctgcgaatcctccggcagagttttcggctacaaagtttgcttcattcatcacccagtatt<br>ccacaggacaagtgagcgtggagattgaatgggagctgcagaaagaaaacagcaaacgctggaatcccgaagtgcagtatacatctaactatgca<br>aaatctgccaacgttgatttcactgtggacaacaatggactttatactgagcctcgccccattggcacccgttacctcacccgtcccctgtaa

AAV7	atggctgccgatggttatcttccagattggctcgaggacaacctctctgagggcattcgcgagtggtgggacctgaaacctggagccccgaaacccaaagccaaccagcaaaagcaggacaacggccggggtctggtgcttcctggctacaagtacctcggacccttcaacggactcgacaagggggagcccgtcaacgcggcggacgcagcggccctcgagcacgacaaggcctacgaccagcagctcaaagcgggtgacaatccgtacctgcggtataaccacgccgacgccgagtttcaggagcgtctgcaagaagatacgtcatttgggggcaacctcgggcgagcagtcttccaggccaagaagcgggttctcgaacctctcggtctggttgaggaaggcgctaagacggctcctgcaaagaagagaccggtagagccgtcacctcagcgttcccccgactcctccacgggcatcggcaagaaaggccagcagcccgccagaaagagactcaatttcggtcagactggcgactcagagtcagtccccgaccctcaacctctcggagaacctccagcagcgccctctagtgtgggatctggtacagtggctgcaggcggtggcgcaccaatggcagacaataacgaaggtgccgacggagtgggtaatgcctcaggaaattggcattgcgattccacatggctgggcgacagagtcattaccaccagcacccgaacctgggccctgcccacctacaacaaccacctctacaagcaaatctccagtgaaactgcaggtagtaccaacgacaacacctacttcggctacagcaccccctgggggtattttgactttaacagattccactgccacttctcaccacgtgactggcagcgactcatcaacaacaactggggattccggcccaagaagctgcggttcaagctcttcaacatccaggtcaaggaggtcacgacgaatgacggcgttacgaccatcgctaataaccttaccagcacgattcaggtattctcggactcggaataccagctgccgtacgtcctcggctctgcgcaccagggctgcctgcctccgttcccggcggacgtcttcatgattcctcagtacggctacctgactctcaacaatggcagtcagtctgtgggacgttcctccttctactgcctggagtacttcccctctcagatgctgagaacgggcaacaactttgagttcagctacagcttcgaggacgtgcctttccacagcagctacgcacacagccagagcctggaccggctgatgaatcccctcatcgaccagtacttgtactacctggccagaacacagagtaacccaggaggcacagctggcaatcgggaactgcagttttaccagggcgggccttcaactatggccgaacaagccaagaattggttacctggaccttgcttccggcaacaaagagtctccaaaacgctggatcaaaacaacaacagcaactttgcttggactggtgccaccaaatatcacctgaacggcagaaactcgttggttaatcccggcgtcgccatggcaactcacaaggacgacgaggaccgctttttcccatccagcggagtcctgatttttggaaaaactggagcaactaacaaaactacattggaaaatgtgttaatgacaaatgaagaagaaattcgtcctactaatcctgtagccacggaagaatacgggatagtcagcagcaacttacaagcggctaatactgcagcccagacacaagttgtcaacaaccagggagccttacctggcatggtctggcagaaccgggacgtgtacctgcagggtcccatctgggccaagattcctcacacggatggcaactttcacccgtctcctttgatgggcggctttggacttaaacatccgcctcctcagatcctgatcaagaacactcccgttcccgctaatcctccggaggtgtttactcctgccaagtttgcttcgttcatcacacagtacagcaccggacaagtcagcgtggaaatcgagtgggagctgcagaaggaaaacagcaagcgctggaacccggagattcagtacacctccaactttgaaaagcagactggtgtggactttgccgttgacagccagggtgtttactctgagcctcgccctattggcactcgttacctcacccgtaatctgtaa
AAV8	atggctgccgatggttatcttccagattggctcgaggacaacctctctgagggcattcgcgagtggtgggcgctgaaacctggagccccgaagcccaaagccaaccagcaaaagcaggacgacggccggggtctggtgcttcctggctacaagtacctcggacccttcaacggactcgacaagggggagcccgtcaacgcggcggacgcagcggccctcgagcacgacaaggcctacgaccagcagctgcaggcgggtgacaatccgtacctgcggtataaccacgccgacgccgagtttcaggagcgtctgcaagaagatacgtcttttgggggcaacctcgggcgagcagtcttccaggccaagaagcgggttctcgaacctctcggtctggttgaggaaggcgctaagacggctcctggaaagaagagaccggtagagccatcaccccagcgttctccagactcctctacgggcatcggcaagaaaggccaacagcccgccagaaaaagactcaattttggtcagactggcgactcagagtcagttccagaccctcaacctctcggagaacctccagcagcgccctctggtgtgggacctaatacaatggctgcaggcggtggcgcaccaatggcagacaataacgaaggcgccgacggagt

	gggtagttcctcgggaaattggcattgcgattccacatggctgggcgacagagtcatcaccaccagcacccgaacctgggccctgcccacctacaa<br>caaccacctctacaagcaaatctccaacgggacatcgggaggagccaccaacgacaacacctacttcggctacagcaccccctgggggtatttt<br>gactttaacagattccactgccacttttcaccacgtgactggcagcgactcatcaacaacaactggggattccggcccaagagactcagcttcaag<br>ctcttcaacatccaggtcaaggaggtcacgcagaatgaaggcaccaagaccatcgccaataacctcaccagcaccatccaggtgtttacggactc<br>ggagtaccagctgccgtacgttctcggctctgcccaccagggctgcctgcctccgttcccggcggacgtgttcatgattccccagtacggctacctaa<br>cactcaacaacggtagtcaggccgtgggacgctcctccttctactgcctggaatactttccttcgcagatgctgagaaccggcaacaacttccagttt<br>acttacaccttcgaggacgtgcctttccacagcagctacgcccacagccagagcttggaccggctgatgaatcctctgattgaccagtacctgtact<br>acttgtctcggactcaaacaacaggaggcacggcaaatacgcagactctgggcttcagccaaggtgggcctaatacaatggccaatcaggcaaag<br>aactggctgccaggaccctgttaccgccaacaacgcgtctcaacgacaaccgggcaaaacaacaatagcaactttgcctggactgctgggacca<br>aataccatctgaatggaagaaattcattggctaatcctggcatcgctatggcaacacacaaagacgacgaggagcgtttttttcccagtaacgggatc<br>ctgatttttggcaaacaaaatgctgccagagacaatgcggattacagcgatgtcatgctcaccagcgaggaagaaatcaaaaccactaaccctgtg<br>gctacagaggaatacggtatcgtggcagataacttgcagcagcaaaacacggctcctcaaattggaactgtcaacagccagggggccttacccggt<br>atggtctggcagaaccgggacgtgtacctgcagggtcccatctgggccaagattcctcacacggacggcaacttccacccgtctccgctgatgggc<br>ggctttggcctgaaacatcctccgcctcagatcctgatcaagaacacgcctgtacctgcggatcctccgaccaccttcaaccagtcaaagctgaac<br>tctttcatcacgcaatacagcaccggacaggtcagcgtggaaattgaatgggagctgcagaaggaaaacagcaagcgctggaaccccgagatcca<br>gtacacctccaactactacaaatctacaagtgtggactttgctgttaatacagaaggcgtgtactctgaaccccgccccattggcacccgttacctca<br>cccgtaatctgtaa
AAV9	atggctgccgatggttatcttccagattggctcgaggacaaccttagtgaaggaattcgcgagtggtgggctttgaaacctggagcccctcaacccaa<br>ggcaaatcaacaacatcaagacaacgctcgaggtcttgtgcttccgggttacaaataccttggacccggcaacggactcgacaagggggagccgg<br>tcaacgcagcagacgcggcggccctcgagcacgacaaggcctacgaccagcagctcaaggccggagacaacccgtacctcaagtacaaccac<br>gccgacgccgagttccaggagcggctcaaagaagatacgtcttttgggggcaacctcgggcgagcagtcttccaggccaaaaagaggcttcttgaa<br>cctcttggtctggttgaggaagcggctaagacggctcctggaaagaagaggcctgtagagcagtctcctcaggaaccggactcctccgcgggtattg<br>gcaaatcgggtgcacagcccgctaaaaagagactcaatttcggtcagactggcgacacagagtcagtcccagaccctcaaccaatcggagaacc<br>tcccgcagccccctcaggtgtgggatctcttacaatggcttcaggtggtggcgcaccagtggcagacaataacgaaggtgccgatggagtgggtagtt<br>cctcgggaaattggcattgcgattcccaatggctgggggacagagtcatcaccaccagcacccgaacctgggccctgcccacctacaacaatcac<br>ctctacaagcaaatctccaacagcacatctggaggatcttcaaatgacaacgcctacttcggctacagcaccccctgggggtattttgacttcaaca<br>gattccactgccacttctcaccacgtgactggcagcgactcatcaacaacaactggggattccggcctaagcgactcaacttcaagctcttcaacat<br>tcaggtcaaagaggttacggacaacaatggagtcaagaccatcgccaataaccttaccagcacggtccaggtcttcacggactcagactatcagct<br>cccgtacgtgctcgggtcggctcacgagggctgcctcccgccgttcccagcggacgttttcatgattcctcagtacgggtatctgacgcttaatgatgg<br>aagccaggccgtgggtcgttcgtccttttactgcctggaatatttcccgtcgcaaatgctaagaacgggtaacaacttccagttcagctacgagtttga<br>gaacgtacctttccatagcagctacgctcacagccaaagcctggaccgactaatgaatccactcatcgaccaatacttgtactatctctcaaagact

	attaacggttctggacagaatcaacaaacgctaaaattcagtgtggccggacccagcaacatggctgtccagggaagaaactacatacctggacc<br>cagctaccgacaacaacgtgtctcaaccactgtgactcaaaacaacaacagcgaatttgcttggcctggagcttcttcttgggctctcaatggacgt<br>aatagcttgatgaatcctggacctgctatggccagccacaaagaaggagaggaccgtttctttcctttgtctggatctttaatttttggcaaacaaggaa<br>ctggaagagacaacgtggatgcggacaaagtcatgataaccaacgaagaagaaattaaaactactaacccggtagcaacggagtcctatggaca<br>agtggccacaaaccaccagagtgcccaagcacaggcgcagaccggctgggttcaaaaccaaggaatacttccgggtatggtttggcaggacaga<br>gatgtgtacctgcaaggacccatttgggccaaaattcctcacacggacggcaactttcacccttctccgctgatgggagggtttggaatgaagcacc<br>cgcctcctcagatcctcatcaaaaacacacctgtacctgcggatcctccaacggccttcaacaaggacaagctgaactctttcatcacccagtatt<br>ctactggccaagtcagcgtggagatcgagtgggagctgcagaaggaaaacagcaagcgctggaacccggagatccagtacacttccaactattac<br>aagtctaataatgttgaatttgctgttaatactgaaggtgtatatagtgaaccccgccccattggcaccagatacctgactcgtaatctgtaa
DJ8	atggctgccgatggttatcttccagattggctcgaggacactctctctgaaggaataagacagtggtggaagctcaaacctggcccaccaccaccaa<br>agcccgcagagcggcataaggacgacagcaggggtcttgtgcttcctgggtacaagtacctcggacccttcaacggactcgacaagggagagccg<br>gtcaacgaggcagacgccgcggccctcgagcacgacaaagcctacgaccggcagctcgacagcggagacaacccgtacctcaagtacaacca<br>cgccgacgccgagttccaggagcggctcaaagaagatacgtcttttgggggcaacctcgggcgagcagtcttccaggccaaaaagaggcttcttga<br>acctcttggtctggttgaggaagcggctaagacggctcctggaaagaagaggcctgtagagcactctcctgtggagccagactcctcctcgggaacc<br>ggaaaggcgggccagcagcctgcaagaaaaagattgaattttggtcagactggagacgcagactcagtcccagaccctcaaccaatcggagaac<br>ctcccgcagccccctcaggtgtgggatctcttacaatggctgcaggcggtggcgcaccaatggcagacaataacgagggcgccgacggagtgggt<br>aattcctcgggaaattggcattgcgattccacatggatgggcgacagagtcatcaccaccagcacccgaacctgggccctgcccacctacaacaa<br>ccacctctacaagcaaatctccaacagcacatctggaggatcttcaaatgacaacgcctacttcggctacagcaccccctgggggtattttgacttt<br>aacagattccactgccacttttcaccacgtgactggcagcgactcatcaacaacaactggggattccggcccaagagactcagcttcaagctcttc<br>aacatccaggtcaaggaggtcacgcagaatgaaggcaccaagaccatcgccaataacctcaccagcaccatccaggtgtttacggactcggagt<br>accagctgccgtacgttctcggctctgcccaccagggctgcctgcctccgttcccggcggacgtgttcatgattccccagtacggctacctaacact<br>caacaacggtagtcaggccgtgggacgctcctccttctactgcctggaatactttccttcgcagatgctgagaaccggcaacaacttccagtttactt<br>acaccttcgaggacgtgcctttccacagcagctacgcccacagccagagcttggaccggctgatgaatcctctgattgaccagtacctgtactactt<br>gtctcggactcaaacaacaggaggcacgacaaatacgcagactctgggcttcagccaaggtgggcctaatacaatggccaatcaggcaaagaac<br>tggctgccaggaccctgttaccgccagcagcgagtatcaaagacatctgcggataacaacaacagtgaatactcgtggactggagctaccaagta<br>ccacctcaatggcagagactctctggtgaatccgggcccggccatggcaagccacaaggacgatgaagaaaagtttttcctcagagcggggttct<br>catctttgggaagcaaggctcagagaaaacaaatgtggacattgaaaaggtcatgattacagacgaagaggaaatcaggacaaccaatcccgtgg<br>ctacggagcagtatggttctgtatctaccaacctccagcaaggcaacacacaagcagctaccgcagatgtcaacacacaaggcgttcttccaggc<br>atggtctggcaggacagagatgtgtaccttcaggggcccatctgggcaaagattccacacacggacggacattttcacccctctcccctcatgggtg<br>gattcggacttaaacaccctccgcctcagatcctgatcaagaacacgcctgtacctgcggatcctccgaccaccttcaaccagtcaaagctgaact<br>ctttcatcacccagtattctactggccaagtcagcgtggagatcgagtgggagctgcagaaggaaaacagcaagcgctggaaccccgagatccagt

	acacctccaactactacaaatctacaagtgtggactttgctgttaatacagaaggcgtgtactctgaaccccgccccattggcacccgttacctcac<br>ccgtaatctgtaa
DJ N589X	atggctgccgatggttatcttccagattggctcgaggacactctctctgaaggaataagacagtggtggaagctcaaacctggcccaccaccaccaa<br>agcccgcagagcggcataaggacgacagcaggggtcttgtgcttcctgggtacaagtacctcggacccttcaacggactcgacaagggagagccg<br>gtcaacgaggcagacgccgcggccctcgagcacgacaaagcctacgaccggcagctcgacagcggagacaacccgtacctcaagtacaacca<br>cgccgacgccgagttccaggagcggctcaaagaagatacgtcttttgggggcaacctcgggcgagcagtcttccaggccaaaaagaggcttcttga<br>acctcttggtctggttgaggaagcggctaagacggctcctggaaagaagaggcctgtagagcactctcctgtggagccagactcctcctcgggaacc<br>ggaaaggcgggccagcagcctgcaagaaaaagattgaattttggtcagactggagacgcagactcagtcccagaccctcaaccaatcggagaac<br>ctcccgcagccccctcaggtgtgggatctcttacaatggctgcaggcggtggcgcaccaatggcagacaataacgagggcgccgacggagtgggt<br>aattcctcgggaaattggcattgcgattccacatggatgggcgacagagtcatcaccaccagcacccgaacctgggccctgcccacctacaacaa<br>ccacctctacaagcaaatctccaacagcacatctggaggatcttcaaatgacaacgcctacttcggctacagcaccccctgggggtattttgacttt<br>aacagattccactgccacttttcaccacgtgactggcagcgactcatcaacaacaactggggattccggcccaagagactcagcttcaagctcttc<br>aacatccaggtcaaggaggtcacgcagaatgaaggcaccaagaccatcgccaataacctcaccagcaccatccaggtgtttacggactcggagt<br>accagctgccgtacgttctcggctctgcccaccagggctgcctgcctccgttcccggcggacgtgttcatgattccccagtacggctacctaacact<br>caacaacggtagtcaggccgtgggacgctcctccttctactgcctggaatactttccttcgcagatgctgagaaccggcaacaacttccagtttactt<br>acaccttcgaggacgtgcctttccacagcagctacgcccacagccagagcttggaccggctgatgaatcctctgattgaccagtacctgtactactt<br>gtctcggactcaaacaacaggaggcacgacaaatacgcagactctgggcttcagccaaggtgggcctaatacaatggccaatcaggcaaagaac<br>tggctgccaggaccctgttaccgccagcagcgagtatcaaagacatctgcggataacaacaacagtgaatactcgtggactggagctaccaagta<br>ccacctcaatggcagagactctctggtgaatccgggcccggccatggcaagccacaaggacgatgaagaaaagtttttcctcagagcggggttct<br>catctttgggaagcaaggctcagagaaaacaaatgtggacattgaaaaggtcatgattacagacgaagaggaaatcaggacaaccaatcccgtgg<br>ctacggagcagtatggttctgtatctaccaacctccagagaggctag
AAV2-retro	atggctgccgatggttatcttccagattggctcgaggacactctctctgaaggaataagacagtggtggaagctcaaacctggcccaccaccaccaa<br>agcccgcagagcggcataaggacgacagcaggggtcttgtgcttcctgggtacaagtacctcggacccttcaacggactcgacaagggagagccg<br>gtcaacgaggcagacgccgcggccctcgagcacgacaaagcctacgaccggcagctcgacagcggagacaacccgtacctcaagtacaacca<br>cgccgacgcggagtttcaggagcgccttaaagaagatacgtcttttgggggcaacctcggacgagcagtcttccaggcgaaaaagagggttcttgaa<br>cctctgggcctggttgaggaacctgttaagacggctccgggaaaaaagaggccggtagagcactctcctgtggagccagactcctcctcgggaacc<br>ggaaaggcgggccagcagcctgcaagaaaaagattgaattttggtcagactggagacgcagactcagtacctgacccccagcctctcggacagcc<br>accagcagccccctctggtctgggaactaatacgatggctacaggcagtggcgcaccaatggcagacaataacgagggcgccgacggagtgggt<br>aattcctcgggaaattggcattgcgattccacatggatgggcgacagagtcatcaccaccagcacccgaacctgggccctgcccacctacaacaa<br>ccacctctacaaacaaatttccagccaatcaggagcctcgaacgacaatcactactttggctacagcaccccttgggggtattttgacttcaacaga<br>ttccactgccacttttcaccacgtgactggcaaagactcatcaacaacaactggggattccgacccaagagactcaacttcaagctctttaacattc

	aagtcaaagaggtcacgcagaatgacggtacgacgacgattgccaataaccttaccagcacggttcaggtgtttactgactcggagtaccagctcc<br>cgtacgtcctcggctcggcgcatcaaggatgcctcccgccgttcccagcagacgtcttcatggtgccacagtatggatacctcaccctgaacgacg<br>ggagtcaggcagtaggacgctcttcattttactgcctggagtactttccttctcagatgctgcgtaccggaaacaactttaccttcagctacacttttgag<br>gacgttcctttccacagcagctacgctcacagccagagtctggaccgtctcatgaatcctctcatcgaccagtacctgtattacttgagcagaacaa<br>acactccaagtggaaccaccacgcagtcaaggcttcagttttctcaggccggagcgagtgacattcgggaccagtctaggaactggcttcctggac<br>cctgttaccgccagcagcgagtatcaaagacatctgcggataacaacaacagtgaatactcgtggactggagctaccaagtaccacctcaatggc<br>agagactctctggtgaatccgggcccggccatggcaagccacaaggacgatgaagaaaagtttttttcctcagagcggggttctcatctttgggaagc<br>aaggctcagagaaaacaaatgtggacattgaaaaggtcatgattacagacgaagaggaaatcaggacaaccaatcccgtggctacggagcagtat<br>ggttctgtatctaccaacctccagagaggcaacctagcagaccaagactacacaaaaactgctaggcaagcagctaccgcagatgtcaacacac<br>aaggcgttcttccaggcatggtctggcaggacagagatgtgtaccttcaggggcccatctgggcaaagattccacacacggacggacattttcaccc<br>ctctcccctcatgggtggattcggacttaaacaccctcctccccagattctcatcaagaacaccccggtacctgcgaatccttcgaccaccttcagt<br>gcggcaaagtttgcttccttcatcacacagtactccacgggacaggtcagcgtggagatcgagtgggagctgcagaaggaaaacagcaaacgctgg<br>aatcccgaaattcagtacacttccaactacaacaagtctattaatgtggactttactgtggacactaatggcgtgtattcagagcctcgccccattggc<br>accagatacctgactcgtaatctgtaa
PHP.S	atggctgccgatggttatcttccagattggctcgaggacaaccttagtgaaggaattcgcgagtggtgggctttgaaacctggagcccctcaacccaa<br>ggcaaatcaacaacatcaagacaacgctagaggtcttgtgcttccgggttacaaataccttggacccggcaacggactcgacaagggggagccgg<br>tcaacgcagcagacgcggcggccctcgagcacgacaaagcctacgaccagcagctcaaggccggagacaacccgtacctcaagtacaaccac<br>gccgacgccgagttccaggagcggctcaaagaagatacgtcttttgggggcaacctcgggcgagcagtcttccaggccaaaaagaggcttcttgaa<br>cctcttggtctggttgaggaagcggctaagacggctcctggaaagaagaggcctgtagagcagtctcctcaggaaccggactcctccgcgggtattg<br>gcaaatcgggtgcacagcccgctaaaaagagactcaatttcggtcagactggcgacacagagtcagtcccagaccctcaaccaatcggagaacc<br>tcccgcagcccccctcaggtgtgggatctcttacaatggcttcaggtggtggcgcaccagtggcagacaataacgaaggtgccgatggagtgggtagtt<br>cctcgggaaattggcattgcgattcccaatggctgggggacagagtcatcaccaccagcacccgaacctgggccctgcccacctacaacaatcac<br>ctctacaagcaaatctccaacagcacatctggaggatcttcaaatgacaacgcctacttcggctacagcaccccctgggggtattttgacttcaaca<br>gattccactgccacttctcaccacgtgactggcagcgactcatcaacaacaactggggattccggcctaagcgactcaacttcaagctctttaacat<br>tcaggtcaaagaggttacggacaacaatggagtcaagaccatcgccaataaccttaccagcacggtccaggtcttcacggactcagactatcagct<br>cccgtacgtgctcgggtcggctcacgagggctgcctcccgccgttcccagcggacgttttcatgattcctcagtacgggtatctgacgcttaatgatgg<br>aagccaggccgtgggtcgttcgtccttttactgcctggaatatttcccgtcgcaaatgctaagaacgggtaacaacttccagttcagctacgagtttga<br>gaacgtacctttccatagcagctacgctcacagccaaagcctggaccgactaatgaatccactcatcgaccaatacttgtactatctctctagaact<br>attaacggttctggacagaatcaacaaacgctaaaattcagtgtggccggacccagcaacatggctgtccagggaagaaactacatacctggacc<br>cagctaccgacaacaacgtgtctcaaccactgtgactcaaaacaacaacagcgaatttgcttggcctggagcttcttcttgggctctcaatggacgt<br>aatagcttgatgaatcctggacctgctatggcctctcacaaagaaggagaggaccgtttctttcctttgtctggatctttaatttttggcaaacaaggtac

	tggcagagacaacgtggatgcggacaaagtcatgataaccaacgaagaagaaattaaaactactaacccggtagcaacggagtcctatggacaa<br>gtggccacaaaccaccagagtgcccaacaggcggttaggacgtctttggcacaggcgcagaccggttgggttcaaaaccaaggaatacttccggg<br>tatggtttggcaggacagagatgtgtacctgcaaggacccatttgggccaaaattcctcacacggacggcaactttcacccttctccgctgatgggag<br>ggtttggaatgaagcacccgcctcctcagatcctcatcaaaaacacacctgtacctgcggatcctccaacggccttcaacaaggacaagctgaac<br>tctttcatcacccagtattctactggtcaagtcagcgtggagatcgagtgggagctgcagaaggaaaacagcaagcgctggaacccggagatccagt<br>acacttccaactattacaagtctaataatgttgaatttgctgttaatactgaaggtgtatatagtgaaccccgccccattggcaccagatacctgactcg<br>taatctgtaa
PHP.eB	atggctgccgatggttatcttccagattggctcgaggacaaccttagtgaaggaattcgcgagtggtgggctttgaaacctggagcccctcaacccaa<br>ggcaaatcaacaacatcaagacaacgctagaggtcttgtgcttccgggttacaaataccttggacccggcaacggactcgacaagggggagccgg<br>tcaacgcagcagacgcggcggccctcgagcacgacaaagcctacgaccagcagctcaaggccggagacaacccgtacctcaagtacaaccac<br>gccgacgccgagttccaggagcggctcaaagaagatacgtcttttgggggcaacctcgggcgagcagtcttccaggccaaaaagaggcttcttgaa<br>cctcttggtctggttgaggaagcggctaagacggctcctggaaagaagaggcctgtagagcagtctcctcaggaaccggactcctccgcgggtattg<br>gcaaatcgggtgcacagcccgctaaaaagagactcaatttcggtcagactggcgacacagagtcagtcccagaccctcaaccaatcggagaacc<br>tcccgcagccccctcaggtgtgggatctcttacaatggcttcaggtggtggcgcaccagtggcagacaataacgaaggtgccgatggagtgggtagtt<br>cctcgggaaattggcattgcgattcccaatggctgggggacagagtcatcaccaccagcacccgaacctgggccctgcccacctacaacaatcac<br>ctctacaagcaaatctccaacagcacatctggaggatcttcaaatgacaacgcctacttcggctacagcaccccctgggggtattttgacttcaaca<br>gattccactgccacttctcaccacgtgactggcagcgactcatcaacaacaactggggattccggcctaagcgactcaacttcaagctctttaacat<br>tcaggtcaaagaggttacggacaacaatggagtcaagaccatcgccaataaccttaccagcacggtccaggtcttcacggactcagactatcagct<br>cccgtacgtgctcgggtcggctcacgagggctgcctcccgccgttcccagcggacgttttcatgattcctcagtacgggtatctgacgcttaatgatgg<br>aagccaggccgtgggtcgttcgtccttttactgcctggaatatttcccgtcgcaaatgctaagaacgggtaacaacttccagttcagctacgagtttga<br>gaacgtacctttccatagcagctacgctcacagccaaagcctggaccgactaatgaatccactcatcgaccaatacttgtactatctctctagaact<br>attaacggttctggacagaatcaacaaacgctaaaattcagtgtggccggacccagcaacatggctgtccagggaagaaactacatacctggacc<br>cagctaccgacaacaacgtgtctcaaccactgtgactcaaaacaacaacagcgaatttgcttggcctggagcttcttcttgggctctcaatggacgt<br>aatagcttgatgaatcctggacctgctatggcctctcacaaagaaggagaggaccgtttctttcctttgtctggatctttaatttttggcaaacaaggtac<br>tggcagagacaacgtggatgcggacaaagtcatgataaccaacgaagaagaaattaaaactactaacccggtagcaacggagtcctatggacaa<br>gtggccacaaaccaccagagtgatgggactttggcggtgccttttaaggcacaggcgcagaccggttgggttcaaaaccaaggaatacttccgggta<br>tggtttggcaggacagagatgtgtacctgcaaggacccatttgggccaaaattcctcacacggacggcaactttcacccttctccgctgatgggaggg<br>tttggaatgaagcacccgcctcctcagatcctcatcaaaaacacacctgtacctgcggatcctccaacggccttcaacaaggacaagctgaactct<br>ttcatcacccagtattctactggtcaagtcagcgtggagatcgagtgggagctgcagaaggaaaacagcaagcgctggaacccggagatccagtac<br>acttccaactattacaagtctaataatgttgaatttgctgttaatactgaaggtgtatatagtgaaccccgccccattggcaccagatacctgactcgta<br>atctgtaa

Sch9	atggctgccgatggttatcttccagattggctcgaggacaacctctctgagggcattcgcgagtggtgggacctgaaacctggagccccgaaaccca<br>aagccaaccagcaaaagcaggacgacggccggggtctggtgcttcctggctacaagtacctcggacccttcaacggactcgacaagggggagcc<br>cgtcaacgcggcggatgcagcggccctcgaacacgacaaggcctacgaccagcagctcaaagcgggtgacaatccgtacctgcggtataacca<br>cgccgacgccgagtttcaggagcgtctgcaagaagatacgtcttttgggggcaacctcgggcgagcagtcttccaggccaagaagagggttctcga<br>acctcttggtctggttgaggaagcggctaagacggctcctggaaagaagaggcctgtagagcagtctcctcaggaaccggactcctccgcgggtatt<br>ggcaaatcgggtgcacagcccgctaaaaagagactcaatttcggtcagactggcgacacagagtcagtcccagaccctcaaccaatcggagaac<br>ctcccgcagccccctcaggtgtgggatctcttacaatggcttcaggtggtggcgcaccagtggcagacaataacgaaggtgccgatggagtgggtag<br>ttcctcgggaaattggcattgcgattcccaatggctgggggacagagtcatcaccaccagcacccgaacctgggccctgcccacctacaacaatc<br>acctctacaagcaaatctccaacagcacatctggaggatcttcaaatgacaacgcctacttcggctacagcaccccctgggggtattttgactttaa<br>cagattccactgccacttttcaccacgtgactggcagcgactcatcaacaacaactggggattccggcccaagagactcagcttcaagctcttcaa<br>catccaggtcaaggaggtcacgcagaatgaaggcaccaagaccatcgccaataacctcaccagcaccatccaggtctttacggactcagactat<br>cagctcccgtacgtgctcgggtcggctcacgagggctgcctcccgccgttcccagcggacgttttcatgattcctcagtacgggtatctgacgcttaat<br>gatggaagccaggccgtgggtcgttcgtccttttactgcctggaatatttcccgtcgcaaatgctaagaacgggtaacaacttccagttcagctacgag<br>tttgagaacgtacctttccatagcagctacgctcacagccaaagcctggaccgactaatgaatccactcatcgaccaatacttgtactatctctcaaa<br>gactattaacggttctggacagaatcaacaaacgctaaaattcagtgtggccggacccagcaacatggctgtccagggaagaaactggcttcctgg<br>accctgttaccgccagcagcgagtatcaaagacatctgcggataacaacaacagtgaatactcgtggactggagctaccaagtaccacctcaatg<br>gcagagactctctggtgaatccgggcccggccatggcaagccacaaggacgatgaagaaaagtttttcctcagagcggggttctcatctttgggaa<br>gcaaggctcagagaaaacaaatgtggacattgaaaaggtcatgattacagacgaagaggaaatcaggacaaccaatcccgtggctacggagcag<br>tatggttctgtatctaccaacctccagagaggcaacagacaagcagctaccgcagatgtcaacacacaaggcgttcttccaggcatggtctggcag<br>gacagagacgtgtacctgcaaggacccatttgggccaaaattcctcacacggacggcaactttcacccttctccgctgatgggagggtttggaatga<br>agcacccgcctcctcagatcctcatcaaaaacacacctgtacctgcggatcctccaacggccttcaacaaggacaagctgaactctttcatcacc<br>cagtattctactggccaagtcagcgtggagatcgagtgggagctgcagaaggaaaacagcaagcgctggaacccggagatccagtacacttccaa<br>ctattacaagtctaataatgttgaatttgctgttaatactgaaggtgtatatagtgaaccccgccccattggcaccagatacctgactcgtaatctgtaa
rh10	atggctgccgatggttatcttccagattggctcgaggacaacctctctgagggcattcgcgagtggtgggacttgaaacctggagccccgaaaccca<br>aagccaaccagcaaaagcaggacgacggccggggtctggtgcttcctggctacaagtacctcggacccttcaacggactcgacaagggggagcc<br>cgtcaacgcggcggacgcagcggccctcgagcacgacaaggcctacgaccagcagctcaaagcgggtgacaatccgtacctgcggtataacca<br>cgccgacgccgagtttcaggagcgtctgcaagaagatacgtcttttgggggcaacctcgggcgagcagtcttccaggccaagaagcgggttctcga<br>acctctcggtctggttgaggaaggcgctaagacggctcctggaaagaagagaccggtagagccatcaccccagcgttctccagactcctctacggg<br>catcggcaagaaaggccagcagcccgcgaaaaagagactcaactttgggcagactggcgactcagagtcagtgcccgaccctcaaccaatcgg<br>agaacccccgcaggcccctctggtctgggatctggtacaatggctgcaggcggtggcgctccaatggcagacaataacgaaggcgccgacggag<br>tgggtagttcctcaggaaattggcattgcgattccacatggctgggcgacagagtcatcaccaccagcacccgaacctgggccctccccacctaca

	acaaccacctctacaagcaaatctccaacgggacttcgggaggaagcaccaacgacaacacctacttcggctacagcacccccctgggggtatttt<br>gactttaacagattccactgccacttctcaccacgtgactggcagcgactcatcaacaacaactggggattccggcccaagagactcaacttcaag<br>ctcttcaacatccaggtcaaggaggtcacgcagaatgaaggcaccaagaccatcgccaataaccttaccagcacgattcaggtctttacggactcg<br>gaataccagctcccgtacgtcctcggctctgcgcaccagggctgcctgcctccgttcccggcggacgtcttcatgattcctcagtacgggtacctgac<br>tctgaacaatggcagtcaggccgtgggccgttcctccttctactgcctggagtactttccttctcaaatgctgagaacgggcaacaactttgagttcag<br>ctaccagtttgaggacgtgccttttcacagcagctacgcgcacagccaaagcctggaccggctgatgaacccccctcatcgaccagtacctgtacta<br>cctgtctcggactcagtccacgggaggtaccgcaggaactcagcagttgctattttctcaggccgggcctaataacatgtcggctcaggccaaaaac<br>tggctacccgggccctgctaccggcagcaacgcgtctccacgacactgtcgcaaaataacaacagcaactttgcctggaccggtgccaccaagta<br>tcatctgaatggcagagactctctggtaaatcccggtgtcgctatggcaacccacaaggacgacgaagagcgatttttttccgtccagcggagtcttaa<br>tgtttgggaaacagggagctggaaaagacaacgtggactatagcagcgttatgctaaccagtgaggaagaaattaaaaccaccaacccagtggcc<br>acagaacagtacggcgtggtggccgataacctgcaacagcaaaacgccgctcctattgtaggggccgtcaacagtcaaggagccttacctggcat<br>ggtctggcagaaccgggacgtgtacctgcagggtcctatctgggccaagattcctcacacggacggaaactttcatccctcgccgctgatgggaggc<br>tttggactgaaacacccgcctcctcagatcctgattaagaatacacctgttcccgcggatcctccaactaccttcagtcaagctaagctggcgtcgtt<br>catcacgcagtacagcaccggacaggtcagcgtggaaattgaatgggagctgcagaaagaaaacagcaaacgctggaacccagagattcaatac<br>acttccaactactacaaatctacaaatgtggactttgctgttaacacagatggcacttattctgagcctcgccccatcggcacccgttacctcacccg<br>taatctgtaa
ITR-CAG-eGFP-WPRE-hGH polyA-ITR sequence (modified from Addgene #37825; CAG promoter underlined)	cgcgctcgctcgctcactgaggccgcccgggcaaagcccgggcgtcgggcgacctttggtcgcccggcctcagtgagcgagcgagcgcgcagag<br>agggagtggccaactccatcactaggggttccttgtagttaatgattaacccgccatgctacttatctacgtagccatgctctaggaagagtaccattg<br>acgtcaataatgacgtatgttcccatagtaacgccaatagggactttccattgacgtcaatgggtggagtatttacggtaaactgcccacttggcagta<br>catcaagtgtatcatatgccaagtacgccccctattgacgtcaatgacggtaaatggcccgcctggcattatgcccagtacatgaccttatgggacttt<br>cctacttggcagtacatctacgtattagtcatcgctattaccatggtcgaggtgagccccacgttctgcttcactctccccatctcccccccctccccac<br>ccccaattttgtatttatttattttttaattattttgtgcagcgatgggggcgggggggggggggggggcgcgcgccaggcggggcggggcggggcgaggg<br>gcggggcggggcgaggcggagaggtgcggcggcagccaatcagagcggcgcgctccgaaagtttccttttatggcgaggcggcggcggcggcggc<br>cctataaaaagcgaagcgcgcggcgggcgggagtcgctgcgcgctgccttcgccccgtgccccgctccgccgccgcctcgcgccgcccgccccg<br>gctctgactgaccgcgttactcccacaggtgagcgggcgggacggcccttctcctccgggctgtaattagcgcttggtttaatgacggcttgtttctttc<br>tgtggctgcgtgaaagccttgaggggctccgggagggccctttgtgcggggggagcggctcggggctgtccgcggggggacggctgccttcggggg<br>gacggggcagggcggggttcggcttctggcgtgtgaccggcggctctagagcctctgctaaccatgttcatgccttcttctttttcctacagctcctggg<br>caacgtgctggttattgtgctgtctcatcattttggcaaagaattggatccgccaccatggtgagcaagggcgaggagctgttcaccggggtggtgccc<br>atcctggtcgagctggacggcgacgtaaacggccacaagttcagcgtgtccggcgagggcgagggcgatgccacctacggcaagctgaccctgaa<br>gttcatttgcaccaccggcaagctgcccgtgccctggcccaccctcgtgaccaccctgacctacggcgtgcagtgcttcagccgctaccccgacc<br>acatgaagcagcacgacttcttcaagtccgccatgcccgaaggctacgtccaggagcgcaccatcttcttcaaggacgacggcaactacaagacc

	cgcgccgaggtgaagttcgagggcgacaccctggtgaaccgcatcgagctgaagggcatcgacttcaaggaggacggcaacatcctggggcaca<br>agctggagtacaactacaacagccacaacgtctatatcatggccgacaagcagaagaacggcatcaaggtgaacttcaagatccgccacaacat<br>cgaggacggcagcgtgcagctcgccgaccactaccagcagaacacccccatcggcgacggccccgtgctgctgcccgacaaccactacctgag<br>cacccagtccgccctgagcaaagaccccaacgagaagcgcgatcacatggtcctgctggagttcgtgaccgccgccgggatcactctcggcatgg<br>acgagctgtacaagtaagaattcgatatcaagcttatcgataatcaacctctggattacaaaatttgtgaaagattgactggtattcttaactatgttgct<br>ccttttacgctatgtggatacgctgctttaatgcctttgtatcatgctattgcttcccgtatggctttcattttctcctccttgtataaatcctggttgctgtctct<br>ttatgaggagttgtggcccgttgtcaggcaacgtggcgtggtgtgcactgtgtttgctgacgcaacccccactggttggggcattgccaccacctgtca<br>gctcctttccgggactttcgctttccccctccctattgccacggcggaactcatcgccgcctgccttgcccgctgctggacaggggctcggctgttggg<br>cactgacaattccgtggtgttgtcggggaaatcatcgtcctttccttggctgctcgcctgtgttgccacctggattctgcgcgggacgtccttctgctacgt<br>cccttcggccctcaatccagcggaccttccttcccgcggcctgctgccggctctgcggcctcttccgcgtcttcgccttcgccctcagacgagtcgga<br>tctccctttgggccgcctccccgtatcgataccgtcgacccgggcggccgcttcgagcagacatgagggtggcatccctgtgacccctccccagtgc<br>ctctcctggccctggaagttgccactccagtgcccaccagccttgtcctaataaaattaagttgcatcattttgtctgactaggtgtccttctataatatt<br>atggggtggaggggggtggtatggagcaaggggcaagttgggaagacaacctgtagggcctgcggggtctattgggaaccaagctggagtgcagtgg<br>cacaatcttggctcactgcaatctccgcctcctgggttcaagcgattctcctgcctcagcctcccgagttgttgggattccaggcatgcatgaccaggc<br>tcagctaattttgtttttttggtagagacggggtttcaccatattggccaggctggtctccaactcctaatctcaggtgatctacccaccttggcctccca<br>aattgctgggattacaggcgtgaaccactgctcccttccctgtccttaacaacaacaattgcattcattttatgtttcaggttcagggggagatgtggga<br>ggtttttaaagcaagtaaaacctctacaaatgtggtaaaatcgataaggatcttcctagagcatggctacgtagataagtagcatggcgggttaatcat<br>taactacaaggaacccctagtgatggagttggccactccctctctgcgcgctcgctcgctcactgaggccgggcgaccaaaggtcgcccgacgcc<br>cgggctttgcccgggcggcctcagtgagcgagcgagcgcgcagctgcattaatgaatcggccaacgcgcggggagaggcggtttgcgtattgggcg<br>ctcttccgcttcctcgctcactgactcgctgcgctcggtcgttcggctgcggcgagcggtatcagctcactcaaaggcggtaatacggttatccacag<br>aatcaggggataacgcaggaaagaacatgtgagcaaaaggccagcaaaaggccaggaaccgtaaaaaggccgcgttgctggcgtttttccatagg<br>ctccgcccccctgacgagcatcacaaaaatcgacgctcaagtcagaggtggcgaaacccgacaggactataaagataccaggcgtttccccctg<br>gaagctccctcgtgcgctctcctgttccgaccctgccgcttaccggatacctgtccgcctttctcccttcgggaagcgtggcgctttctcatagctcac<br>gctgtaggtatctcagttcggtgtaggtcgttcgctccaagctgggctgtgtgcacgaaccccccgttcagcccgaccgctgcgccttatccggtaact<br>atcgtcttgagtccaacccggtaagacacgacttatcgccactggcagcagccactggtaacaggattagcagagcgaggtatgtaggcggtgctac<br>agagttcttgaagtggtggcctaactacggctacactagaagaacagtatttggtatctgcgctctgctgaagccagttaccttcggaaaaagagttgg<br>tagctcttgatccggcaaacaaaccaccgctggtagcggtggtttttttgtttgcaagcagcagattacgcgcagaaaaaaaggatctcaagaagatc<br>ctttgatcttttctacggggtctgacgctcagtggaacgaaaactcacgttaagggattttggtcatgagattatcaaaaaggatcttcacctagatcctt<br>ttaaattaaaaatgaagttttaaatcaatctaaagtatatatgagtaaacttggtctgacagttaccaatgcttaatcagtgaggcacctatctcagcgat<br>ctgtctatttcgttcatccatagttgcctgactccccgtcgtgtagataactacgatacgggagggcttaccatctggccccagtgctgcaatgataccg<br>cgagacccacgctcaccggctccagatttatcagcaataaaccagccagccggaagggccgagcgcagaagtggtcctgcaactttatccgcctc

	catccagtctattaattgttgccgggaagctagagtaagtagttcgccagttaatagtttgcgcaacgttgttgccattgctacaggcatcgtggtgtcac<br>gctcgtcgtttggtatggcttcattcagctccggttcccaacgatcaaggcgagttacatgatcccccatgttgtgcaaaaaagcggttagctccttcgg<br>tcctccgatcgttgtcagaagtaagttggccgcagtgttatcactcatggttatggcagcactgcataattctcttactgtcatgccatccgtaagatgctt<br>ttctgtgactggtgagtactcaaccaagtcattctgagaatagtgtatgcggcgaccgagttgctcttgcccggcgtcaatacgggataataccgcgcc<br>acatagcagaactttaaaagtgctcatcattggaaaacgttcttcggggcgaaaactctcaaggatcttaccgctgttgagatccagttcgatgtaacc<br>cactcgtgcacccaactgatcttcagcatcttttactttcaccagcgtttctgggtgagcaaaaacaggaaggcaaaatgccgcaaaaaagggaata<br>agggcgacacggaaatgttgaatactcatactcttcctttttcaatattattgaagcatttatcagggttattgtctcatgagcggatacatatttgaatgta<br>tttagaaaaataaacaaataggggttccgcgcacatttccccgaaaagtgccacctaaattgtaagcgttaatattttgttaaaattcgcgttaaattttt<br>gttaaatcagctcattttttaaccaataggccgaaatcggcaaaatcccttataaatcaaaagaatagaccgagatagggttgagtgttgttccagtttg<br>gaacaagagtccactattaaagaacgtggactccaacgtcaaagggcgaaaaaccgtctatcagggcgatggcccactacgtgaaccatcaccc<br>taatcaagttttttggggtcgaggtgccgtaaagcactaaatcggaaccctaaagggagcccccgatttagagcttgacggggaaagccggcgaacg<br>tggcgagaaaggaagggaagaaagcgaaaggagcgggcgctagggcgctggcaagtgtagcggtcacgctgcgcgtaaccaccacacccgccg<br>cgcttaatgcgccgctacagggcgcgtcccattcgccattcaggctgcgcaactgttgggaagggcgatcggtgcgggcctcttcgctattacgccag<br>ctg
**AAV1 BC**	CGACGGTCGTTTACCTTGTGGATCA
**AAV2 BC**	CGACGAGTCGTTTACCTTGTGGATC
**AAV5 BC**	CGACGGGATCAGTCGTTTACCTTGT
**AAV6 BC**	CGACGTTACCTTGTGGATCAGTCGT
**AAV7 BC**	CGACGGTTTACCTTGTGGATCAGTC
**AAV8 BC**	CGACGCGTTTACCTTGTGGATCAGT
**AAV9 BC**	CGACGACCTTGTGGATCAGTCGTTT
**DJ8 BC**	CGACGTGGATCAGTCGTTTACCTTG
**DJ BC**	CGACGTTTACCTTGTGGATCAGTCG
**2retro BC**	CGACGCCTTGTGGATCAGTCGTTTA
**PHP.s BC**	CGACGTCAGTCGTTTACCTTGTGGA
**PHP.eB BC**	CGACGGATCAGTCGTTTACCTTGTG
**sch9 BC**	CGACGTCGTTTACCTTGTGGATCAG
**rh10 BC**	CGACGCAGTCGTTTACCTTGTGGAT

Figure S1

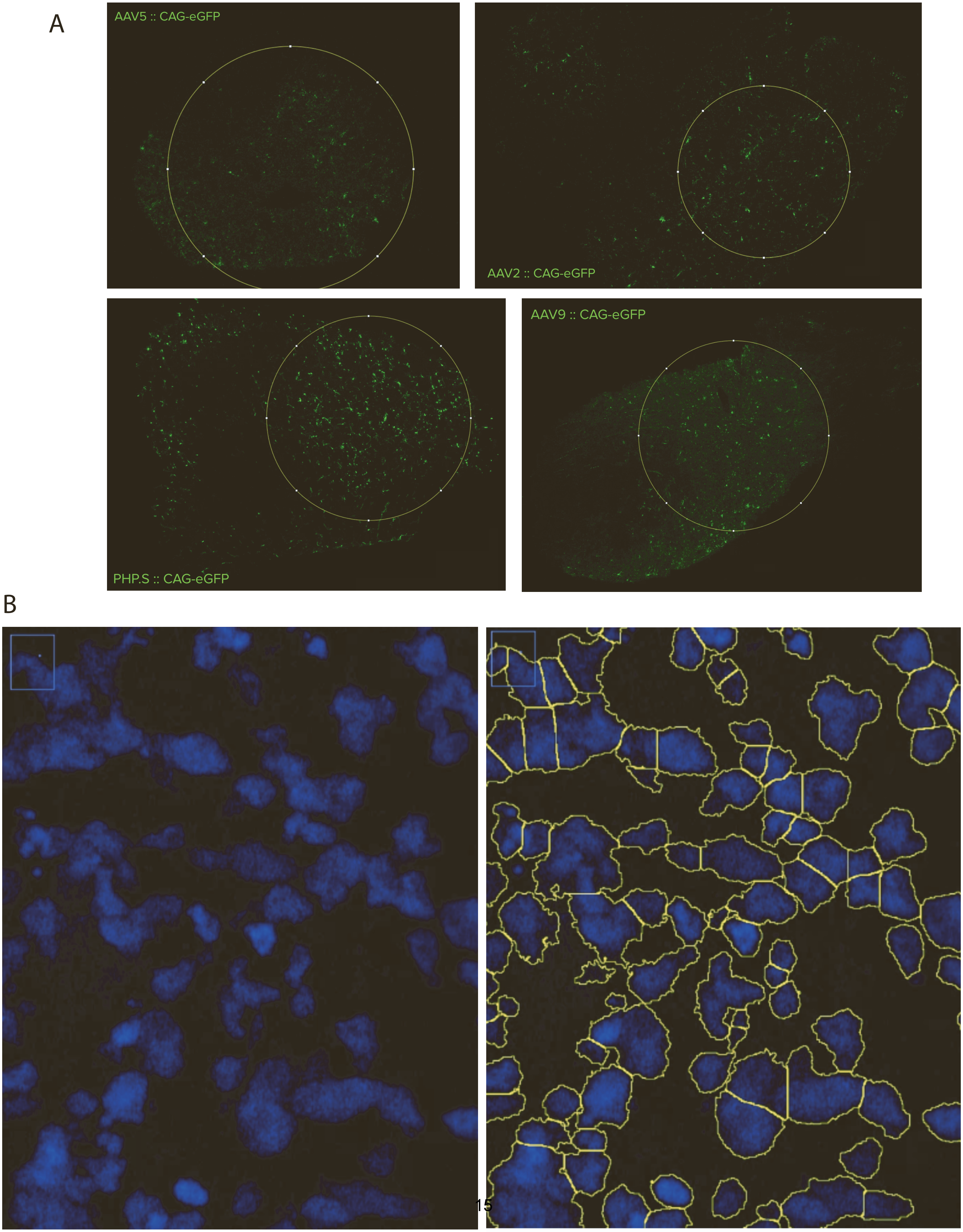

Figure S1 | Selection of regions of interest (ROI) for image analysis. a, Example screenshots of the 2mm circular region used to restrict analysis to the highest 2mm diameter circlular region of each slice. **b,** Representative images showing the custom automated DAPI-centric ROI identification. The average intensity within each ROI is separately calculated for each of the four channels.

Figure S2

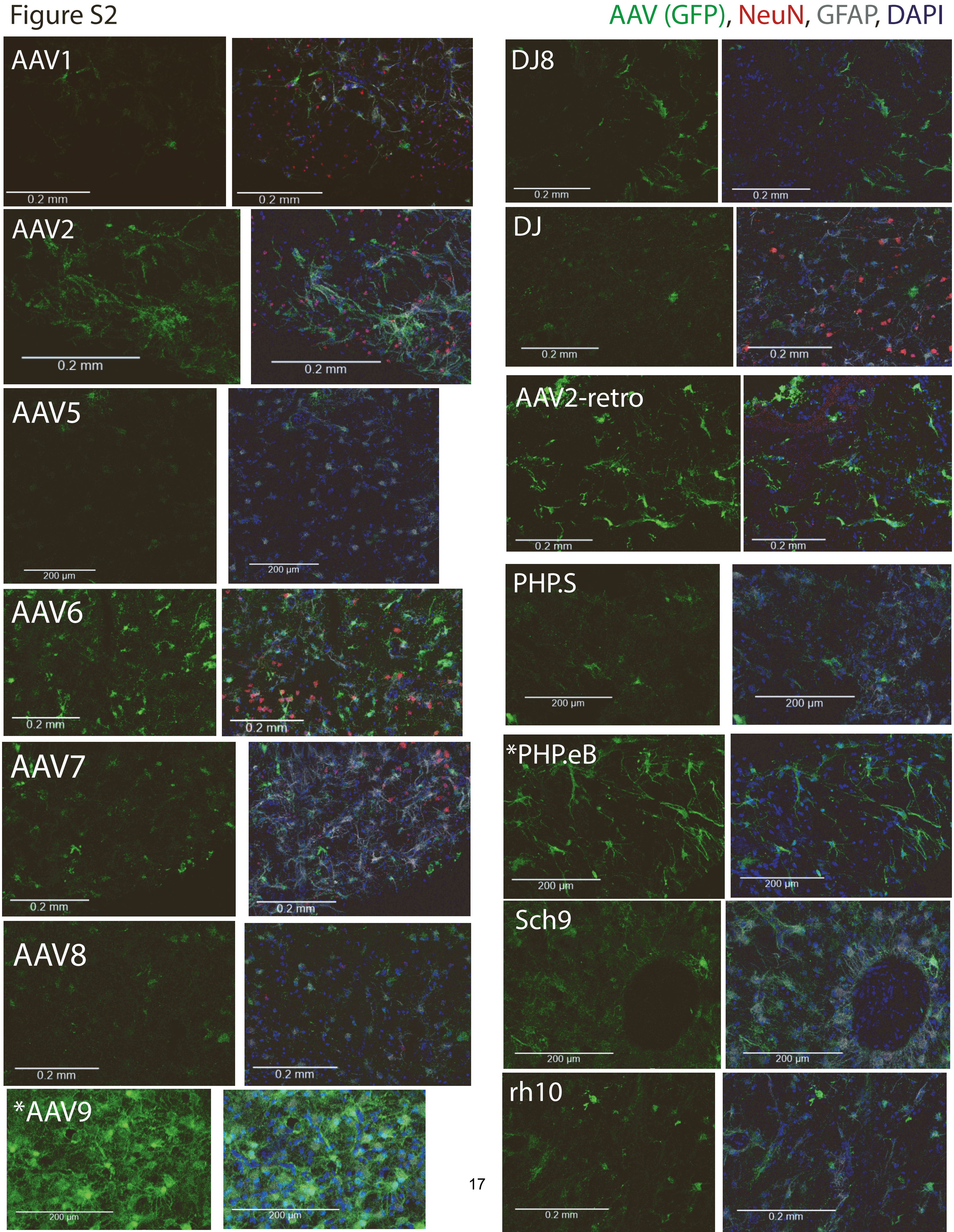
AAV (GFP), NeuN, GFAP, DAPI
AAV1
0.2 mm
0.2 mm
AAV2
0.2 mm
0.2 mm
AAV5
200 µm
200 µm
AAV6
0.2 mm
0.2 mm
AAV7
0.2 mm
0.2 mm
AAV8
0.2 mm
0.2 mm
*AAV9
200 µm
200 µm
DJ8
0.2 mm
0.2 mm
DJ
0.2 mm
0.2 mm
AAV2-retro
0.2 mm
0.2 mm
PHP.S
200 µm
200 µm
*PHP.eB
200 µm
200 µm
Sch9
200 µm
200 µm
rh10
0.2 mm
0.2 mm

Figure S2 | Composite figure showing representative images from each capsid variant.

Figure S3

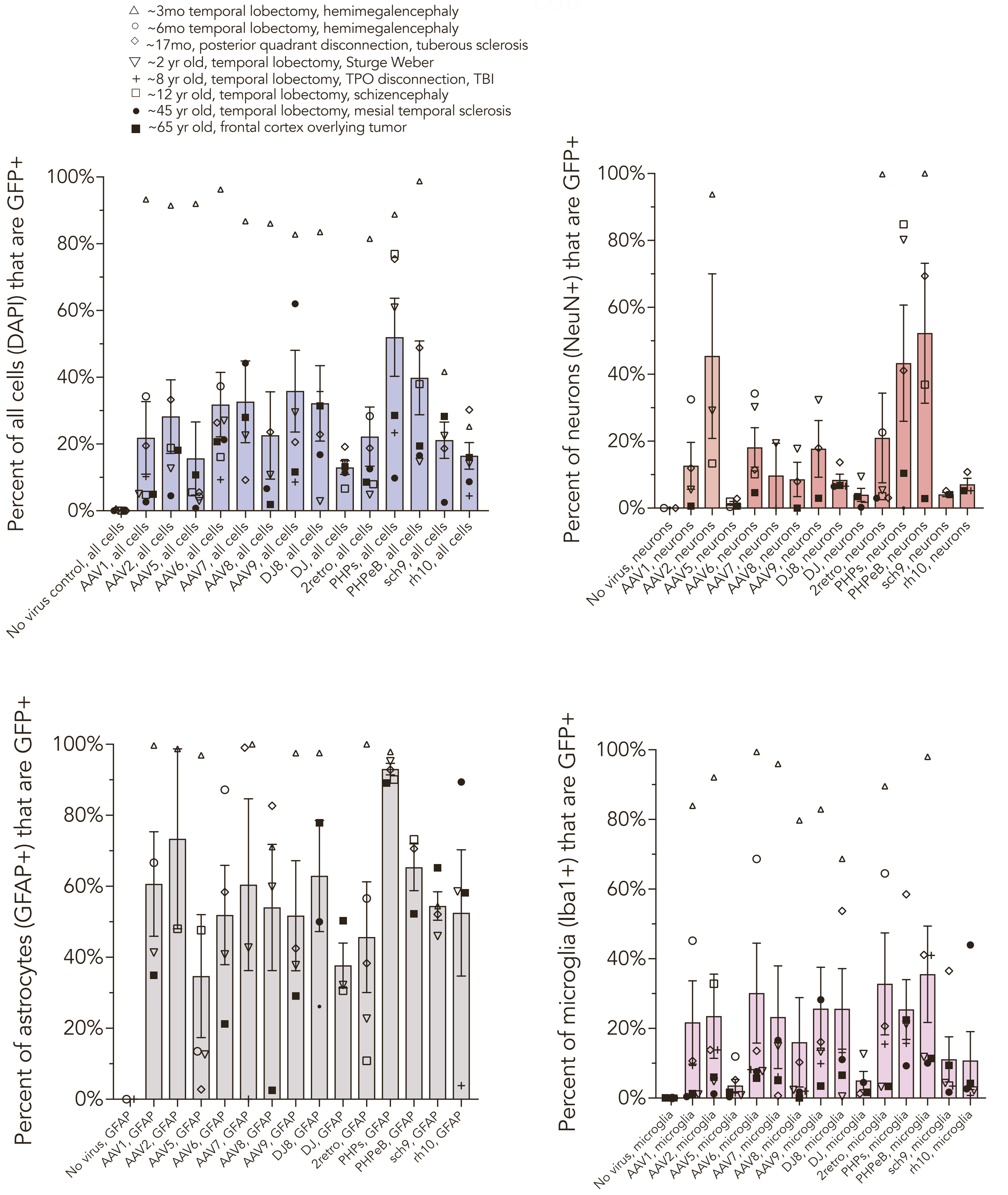

~3mo temporal lobectomy, hemimegalencephaly
~6mo temporal lobectomy, hemimegalencephaly
~17mo, posterior quadrant disconnection, tuberous sclerosis
~2 yr old, temporal lobectomy, Sturge Weber
~8 yr old, temporal lobectomy, TPO disconnection, TBI
~12 yr old, temporal lobectomy, schizencephaly
~45 yr old, temporal lobectomy, mesial temporal sclerosis
~65 yr old, frontal cortex overlying tumor
Percent of all cells (DAPI) that are GFP+
100%
80%
60%
40%
20%
0%
No virus control, all cells
AAV1, all cells
AAV2, all cells
AAV5, all cells
AAV6, all cells
AAV7, all cells
AAV8, all cells
AAV9, all cells
DJ8, all cells
DJ, all cells
2retro, all cells
PHPs, all cells
PHPeB, all cells
sch9, all cells
rh10, all cells
Percent of neurons (NeuN+) that are GFP+
100%
80%
60%
40%
20%
0%
No virus, neurons
AAV1, neurons
AAV2, neurons
AAV5, neurons
AAV6, neurons
AAV7, neurons
AAV8, neurons
AAV9, neurons
DJ8, neurons
DJ, neurons
2retro, neurons
PHPs, neurons
PHPeB, neurons
sch9, neurons
rh10, neurons
Percent of astrocytes (GFAP+) that are GFP+
100%
80%
60%
40%
20%
0%
No virus, GFAP
AAV1, GFAP
AAV2, GFAP
AAV5, GFAP
AAV6, GFAP
AAV7, GFAP
AAV8, GFAP
AAV9, GFAP
DJ8, GFAP
DJ, GFAP
2retro, GFAP
PHPs, GFAP
PHPeB, GFAP
sch9, GFAP
rh10, GFAP
Percent of microglia (Iba1+) that are GFP+
100%
80%
60%
40%
20%
0%
No virus, microglia
AAV1, microglia
AAV2, microglia
AAV5, microglia
AAV6, microglia
AAV7, microglia
AAV8, microglia
AAV9, microglia
DJ8, microglia
DJ, microglia
2retro, microglia
PHPs, microglia
PHPeB, microglia
sch9, microglia
rh10, microglia

Figure S3 | Alternative analysis using the entire imaged tissue slice.

Graphs for each cell type using the alternative analysis that used the entire imaged slice, rather than a 2mm circle centered over the region of greatest transduction.

Figure S4

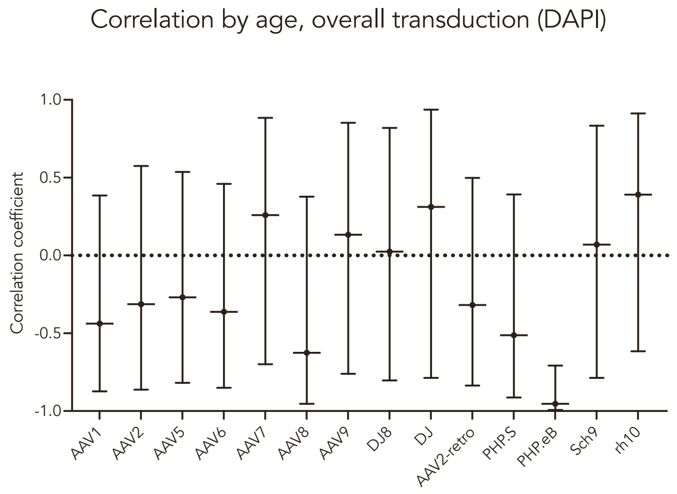
Correlation by age, overall transduction (DAPI)
Correlation coefficient
1.0
0.5
0.0
-0.5
-1.0
AAV1
AAV2
AAV5
AAV6
AAV7
AAV8
AAV9
DJ8
DJ
AAV2-retro
PHP.S
PHP.eB
Sch9
rh10

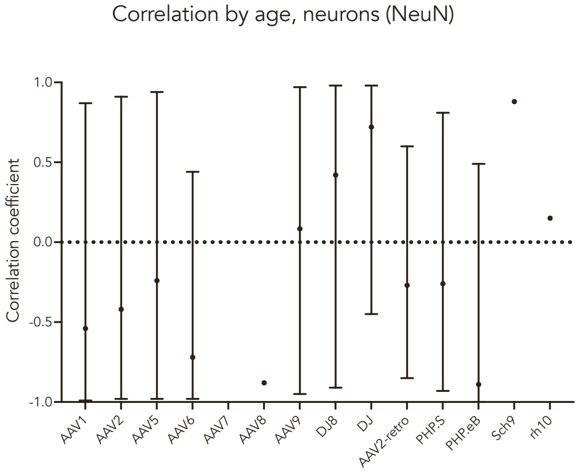
Correlation by age, neurons (NeuN)
Correlation coefficient
1.0
0.5
0.0
-0.5
-1.0
AAV1
AAV2
AAV5
AAV6
AAV7
AAV8
AAV9
DJ8
DJ
AAV2-retro
PHP.S
PHP.eB
Sch9
rh10

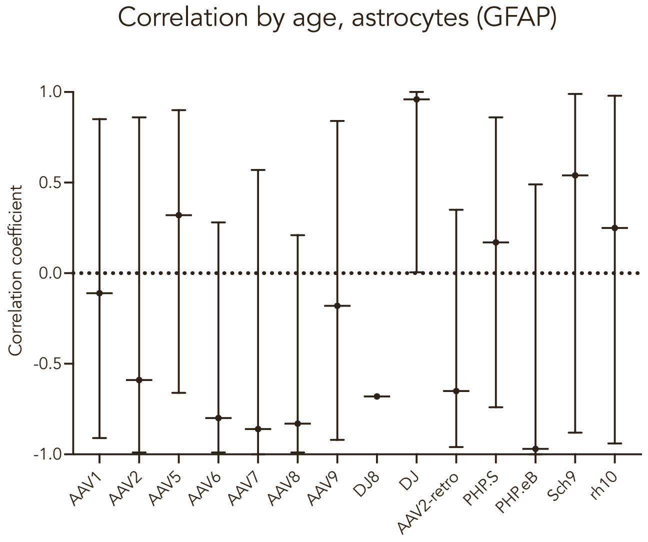
Correlation by age, astrocytes (GFAP)
Correlation coefficient
1.0
0.5
0.0
-0.5
-1.0
AAV1
AAV2
AAV5
AAV6
AAV7
AAV8
AAV9
DJ8
DJ
AAV2-retro
PHP.S
PHP.eB
Sch9
rh10

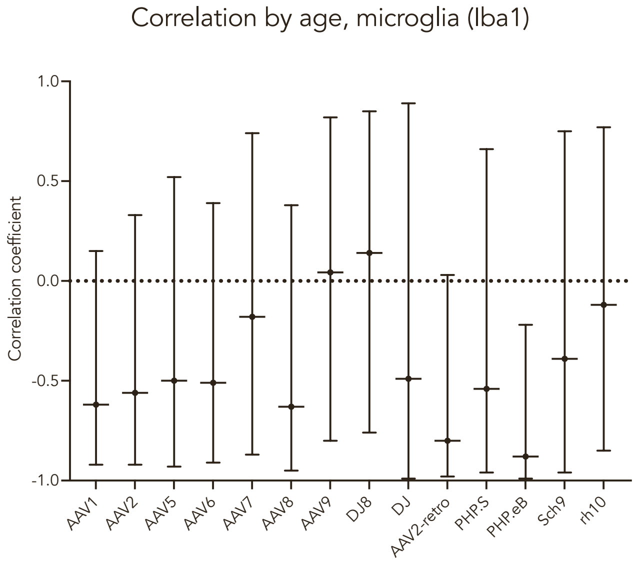
Correlation by age, microglia (Iba1)
Correlation coefficient
1.0
0.5
0.0
-0.5
-1.0
AAV1
AAV2
AAV5
AAV6
AAV7
AAV8
AAV9
DJ8
DJ
AAV2-retro
PHP.S
PHP.eB
Sch9
rh10

Figure S4 | Correlation between age and transduction efficiency, by cell type. Plots showing the estimated correlation by cell type of the relationship between patient age and transduction efficiency across capsid variants.

Figure S5

a.

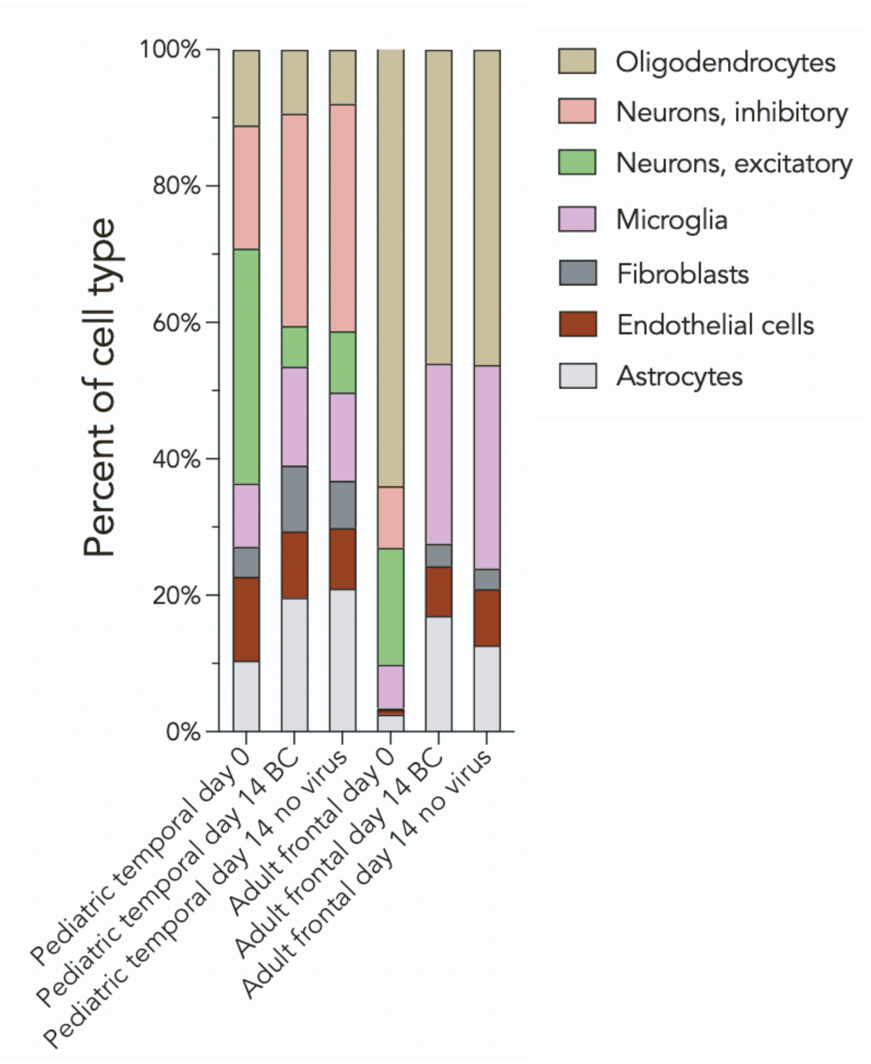

100%
80%
60%
40%
20%
0%
Percent of cell type
Pediatric temporal day 0
Pediatric temporal day 14 BC
Pediatric temporal day 14 no virus
Adult frontal day 0
Adult frontal day 14 BC
Adult frontal day 14 no virus
Oligodendrocytes
Neurons, inhibitory
Neurons, excitatory
Microglia
Fibroblasts
Endothelial cells
Astrocytes

b.

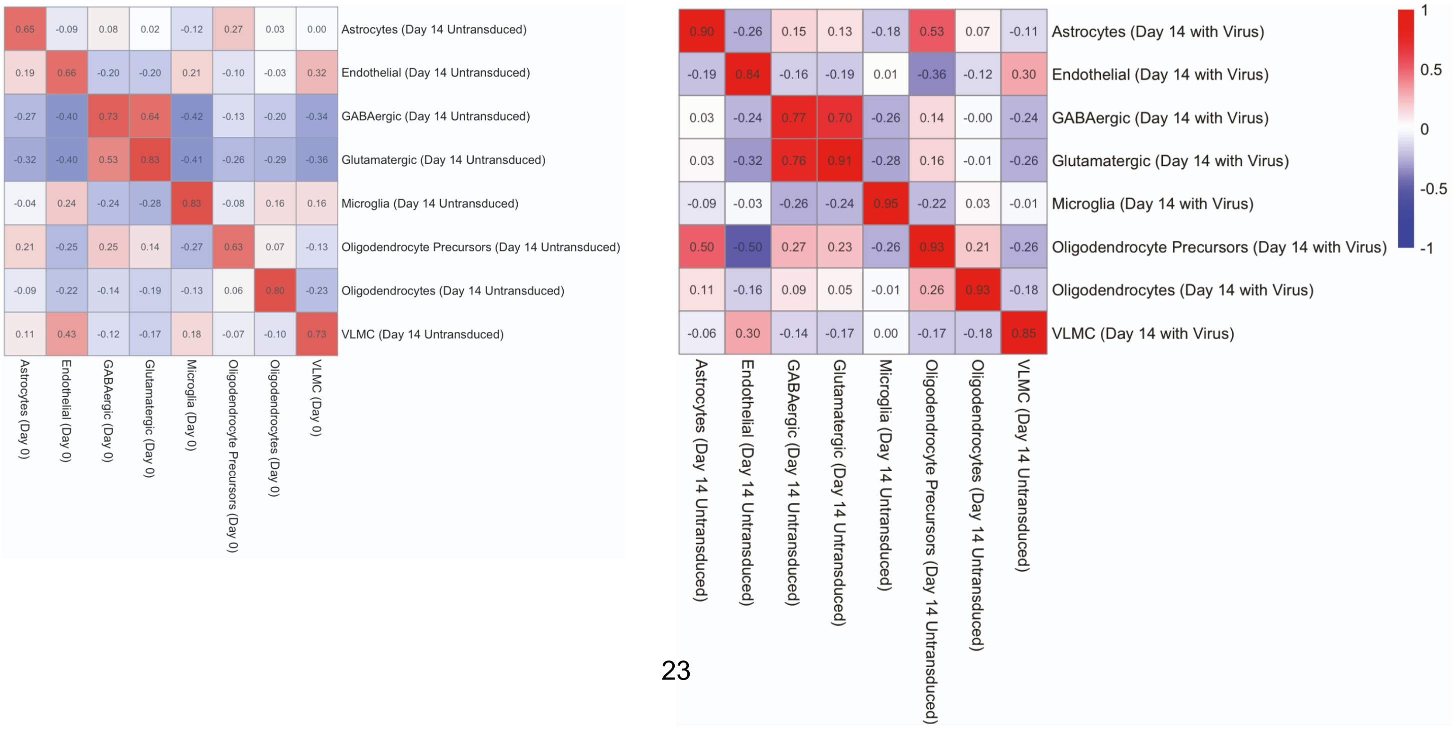

0.65 -0.09 0.08 0.02 -0.12 0.27 0.03 0.00 Astrocytes (Day 14 Untransduced)
0.19 0.66 -0.20 -0.20 0.21 -0.10 -0.03 0.32 Endothelial (Day 14 Untransduced)
-0.27 -0.40 0.73 0.64 -0.42 -0.13 -0.20 -0.34 GABAergic (Day 14 Untransduced)
-0.32 -0.40 0.53 0.83 -0.41 -0.26 -0.29 -0.36 Glutamatergic (Day 14 Untransduced)
-0.04 0.24 -0.24 -0.28 0.83 -0.08 0.16 0.16 Microglia (Day 14 Untransduced)
0.21 -0.25 0.25 0.14 -0.27 0.63 0.07 -0.13 Oligodendrocyte Precursors (Day 14 Untransduced)
-0.09 -0.22 -0.14 -0.19 -0.13 0.06 0.80 -0.23 Oligodendrocytes (Day 14 Untransduced)
0.11 0.43 -0.12 -0.17 0.18 -0.07 -0.10 0.73 VLMC (Day 14 Untransduced)
Astrocytes (Day 0)
Endothelial (Day 0)
GABAergic (Day 0)
Glutamatergic (Day 0)
Microglia (Day 0)
Oligodendrocyte Precursors (Day 0)
Oligodendrocytes (Day 0)
VLMC (Day 0)
0.90 -0.26 0.15 0.13 -0.18 0.53 0.07 -0.11 Astrocytes (Day 14 with Virus)
-0.19 0.84 -0.16 -0.19 0.01 -0.36 -0.12 0.30 Endothelial (Day 14 with Virus)
0.03 -0.24 0.77 0.70 -0.26 0.14 -0.00 -0.24 GABAergic (Day 14 with Virus)
0.03 -0.32 0.76 0.91 -0.28 0.16 -0.01 -0.26 Glutamatergic (Day 14 with Virus)
-0.09 -0.03 -0.26 -0.24 0.95 -0.22 0.03 -0.01 Microglia (Day 14 with Virus)
0.50 -0.50 0.27 0.23 -0.26 0.93 0.21 -0.26 Oligodendrocyte Precursors (Day 14 with Virus)
0.11 -0.16 0.09 0.05 -0.01 0.26 0.93 -0.18 Oligodendrocytes (Day 14 with Virus)
-0.06 0.30 -0.14 -0.17 0.00 -0.17 -0.18 0.85 VLMC (Day 14 with Virus)
Astrocytes (Day 14 Untransduced)
Endothelial (Day 14 Untransduced)
GABAergic (Day 14 Untransduced)
Glutamatergic (Day 14 Untransduced)
Microglia (Day 14 Untransduced)
Oligodendrocyte Precursors (Day 14 Untransduced)
Oligodendrocytes (Day 14 Untransduced)
VLMC (Day 14 Untransduced)
1
0.5
0
-0.5
-1

Figure S5 | Cell type composition changes across fourteen days. a, Proportions of cell types present in sequenced tissues at each time point, with and without virus. **b**, Correlation matrices (correlation calculated across all differentially expressed genes identified for that sample's clusters) for day 0 versus day 14 without virus, and for day 14 with virus versus day 14 without virus. Pearson correlation coefficients are printed within each cell.

Figure S6

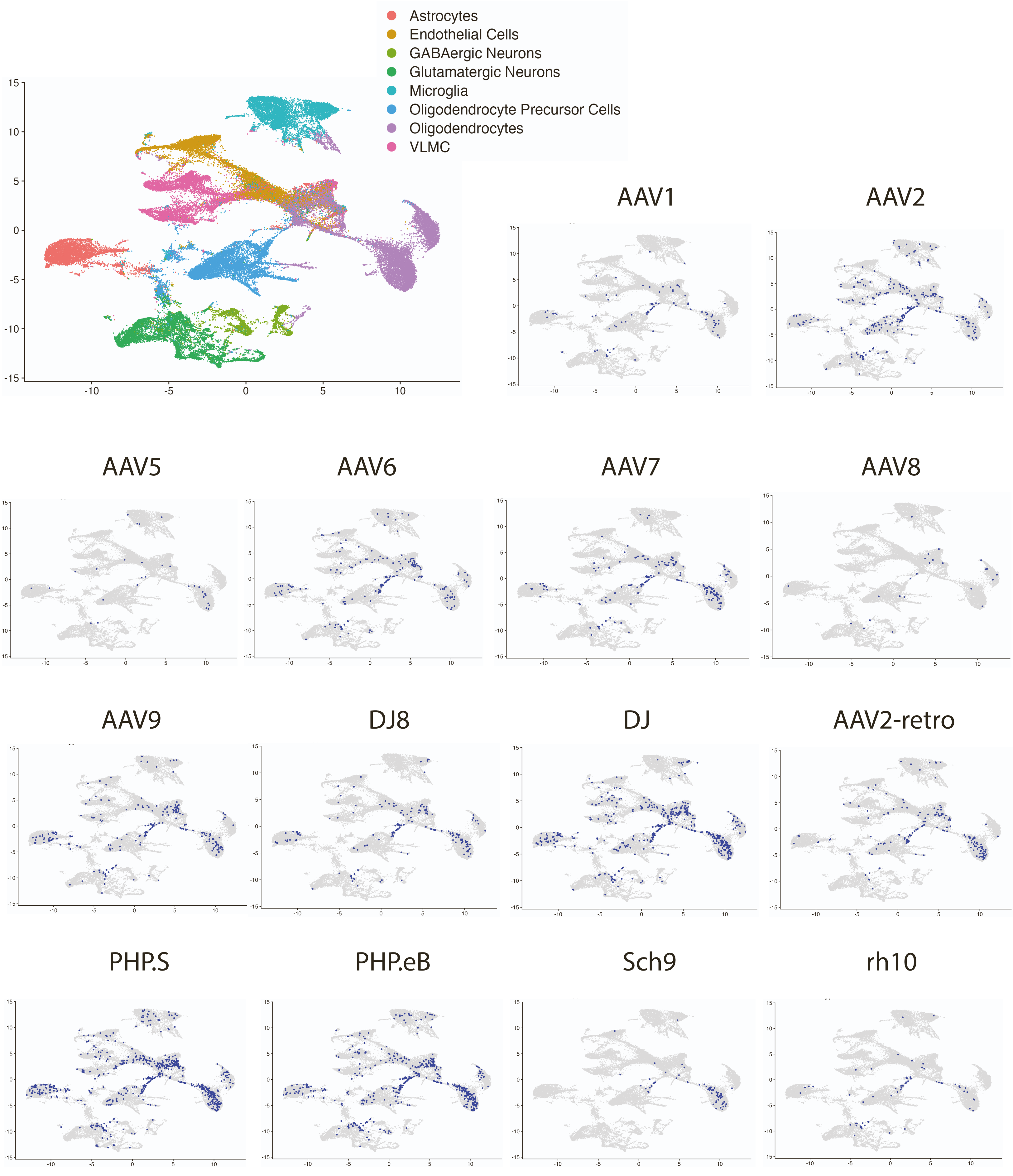
Astrocytes
Endothelial Cells
GABAergic Neurons
Glutamatergic Neurons
Microglia
Oligodendrocyte Precursor Cells
Oligodendrocytes
VLMC
AAV1
AAV2
AAV5
AAV6
AAV7
AAV8
AAV9
DJ8
DJ
AAV2-retro
PHP.S
PHP.eB
Sch9
rh10

Figure S6 | Transduction of cell clusters by AAV capsid variants.

UMAP plots are shown for each AAV capsid variant, with transduced cells appearing dark blue. For all variants, cells are largely scattered across all cell clusters, indicated a lack of specificity for any certain cell type.
