# Supplementary material for: Common AAV gene therapy vectors show nonselective transduction of ex vivo human brain tissue
Source: Mol Ther Methods Clin Dev. 2025 May 21;33(2):101494. doi: 10.1016/j.omtm.2025.101494 (PMC12169722; doi:10.1016/j.omtm.2025.101494)
Supplement: Table S2. Estimated AAV dosing per mg of brain tissue in intraparenchymal, convection-enhanced delivery trials — For clinical trials that have published their vector doses and volume infused, we assume the 1:1 to 1:3 infusate volume to brain volume infused that has been reported, in order to estimate the number of viruses per mg of brain tissue. [file mmc2.pdf]

Table S2

Trial	NCT	Low estimate, vector genomes per mg brain infused*	High vector genomes per mg brain infused**
NTN Phase 1a, 2a	NCT00252850	1.05E+09	3.16E+09
NTN Phase 1b, 2b	NCT00252850	9.71E+08	2.91E+09
VY-AADC01 phase 1 UCSF cohort 1	NCT01973543	5.39E+08	1.62E+09
VY-AADC01 phase 1 UCSF cohort 2	NCT01973543	5.39E+08	1.62E+09
VY-AADC01 phase 1 UCSF cohort 3	NCT01973543	1.69E+09	5.07E+09
GAD phase 1 2007 Lancet low dose	NCT00195143	4.47E+08	1.34E+09
GAD phase 1 2007 Lancet mid dose	NCT00195143	1.34E+09	4.02E+09
GAD phase 1 2007 Lancet high dose	NCT00195143	4.47E+09	1.34E+10
GAD phase 2	NCT00643890	3.24E+07	9.71E+07
Tay Sachs Phase 1/2 abstract CNS 2023	NCT04669535	1.06E+10	3.17E+10
NTN Sangamo, AAV2-NRTN in PD	NCT00985517	2.16E+09	6.47E+09
MPS, rh.10, SGSH	NCT03612869	3.24E+08	9.71E+08
**Median**		**1.01E+09**	**3.03E+09**
Mean		2.01E+09	6.03E+09

*Low estimate of vg/mg tissue: (#vector genomes infused)/(uL of infusate * 3 * 1.03mg)
**High estimate of vg/mg tissue: (#vector genomes infused)/(uL of infusate * 1 * 1.03mg)
